# Multi-Component Syntheses of Spiro[furan-2,3′-indoline]-3-carboxylate Derivatives Using Ionic Liquid Catalysts

**DOI:** 10.3390/molecules29061223

**Published:** 2024-03-08

**Authors:** Mehdi Khalaj, Maryam Zarandi, Malihe Samadi Kazemi, Seyed Mahmoud Musavi, Johannes Hohnsen, Axel Klein

**Affiliations:** 1 Department of Chemistry, Buinzahra Branch, Islamic Azad University, Buinzahra 1477893855, Iran; maryam.zarandi88@gmail.com (M.Z.); smms110@yahoo.com (S.M.M.); 2 Department of Chemistry, Faculty of Science, Bojnourd Branch, Islamic Azad University, Bojnourd 9417697796, Iran; samadi24243@yahoo.com; 3 Faculty of Mathematics and Natural Sciences, Department of Chemistry and Biochemistry, Institute for Inorganic Chemistry, University of Cologne, Greinstrasse 6, D-50939 Köln, Germany; jhohnsen@smail.uni-koeln.de

**Keywords:** spiro[furan-2,3′-indoline]-3-carboxylate derivatives, ionic liquid catalyst, butenolides, spiro compounds, imidazolium hydrogen sulfate, pyridinium hydrogen sulfate

## Abstract

Two previously described Brønsted acidic ionic liquids, 3,3′-(1,6-hexanediyl)bis(1-methyl)-1*H*-imidazolium hydrogen sulfate (**Cat1**) and 1,1′-(1,6-hexanediyl)bis(pyridinium) hydrogen sulfate (**Cat2**), were used as catalysts for the preparation of spiro[furan-2,3′-indoline]-3-carboxylate derivatives via a three-component reaction of anilines, isatins (*N*-alkyl-indoline-2,3-diones), and diethyl acetylenedicarboxylate, in high yields. The use of ultrasonic (US) irradiation led to the targeted products (**1a**–**15a**) in high yields ranging from 80% to 98%. Under the same conditions, the use of sulfuric acid and acetic acid as a Brønstedt catalyst did not yield the desired benchmark product **1a**.

## 1. Introduction

The use of ionic liquids (ILs) as organo-catalysts has recently received considerable attention [1,2,3,4,5]. More specifically, Brønsted acidic ILs are easy-to-make and versatile catalysts [1,2,3,4,5,6,7,8].

**Scheme 1:**
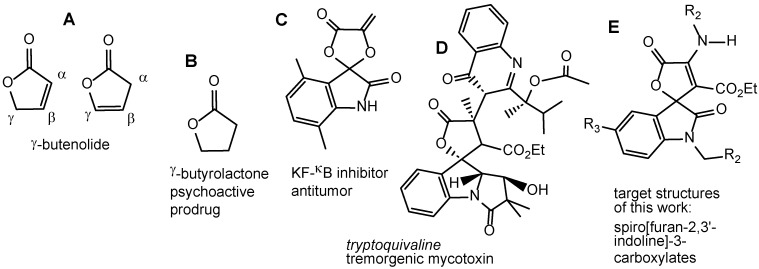
(**A**) basic γ-butenolide structures. (**B**) γ-butyrolactone. (**C**) a spiro-cyclic furan-indoline KF-κB inhibitor. (**D**) *tryptoquivaline*, a mycotoxin (**E**) Target structures of this work.

The furan-2(5*H*)-one cores, generally named butenolides, have been known for their interesting chemical, biological, and medical properties (Scheme 1) [9,10,11]. In particular, spirocyclic butanolide derivatives have recently come into the focus of interest [12,13,14,15,16]. Thus, the development of synthetic methods for butenolides is of great importance. Amongst them are formylation at the ortho position of aromatic carboxylic acids and its derivatives [17,18], [3+2] annulation reactions between electrophilic carbonyls and homoenolate nucleophiles [12,13,14,15,16], ring-opening [3+2] annulation reactions of cyclopropenones with amides [19], Ph_3_P catalyzed ring-opening of functionalized cyclopropenones [20], base-controlled azirine ring expansion [21], Baeyer–Villiger oxidation of 3-arylcyclobutanones [9,22], and Passerini-like three-component condensation reactions [23]. Further routes include Aldol reactions, asymmetric Aldol reaction of silyloxyfurans, asymmetric Mannich reaction, asymmetric Mukaiyama–Michael reaction of silyloxyfurans, and enantioselective radical oxyfunctionalizations [9,24,25]. Unfortunately, these procedures frequently suffer from small substrate scopes.

Another simple strategy for the synthesis of such furan-2(5*H*)-one cores is a three-component reaction using acetylene carboxylates, anilines, and carbonyl compounds [9,26,27,28,29,30,31,32,33,34,35,36]. Various materials have been used as catalysts for these kinds of transformations. Amongst them are heterogenous Lewis acid catalysts such as β-cyclodextrin [26], SnCl_2_ [27], SnO nanoparticles [28], ZnO nanoparticles [29], Al-doped ZnO nanostructures [30], and nano-CdZr_4_(PO_4_)_6_ [31], heterogeneous Brønstedt catalysts such as silica-gel-supported polyphosphoric acid (PPA/SiO_2_) [32] and HY zeolite [33], and homogeneous acid catalysts such as Al(HSO_4_)_3_ [34], tetra-*n*-butylammonium hydrogen sulfate [35], and *N*-methyl-2-pyrrolidonium hydrogen sulfate [36].

**Scheme 2:**
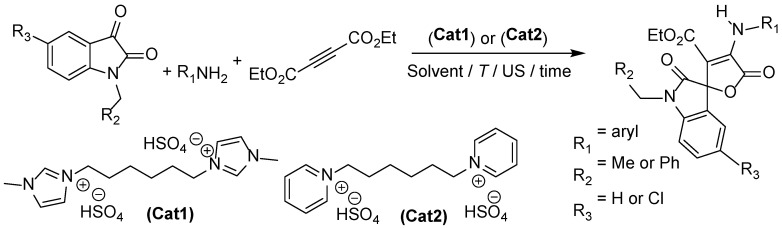
Syntheses of 2′,5-dioxo-5*H*-spiro[furan-2,3′-indoline]-3-carboxylate derivatives using 1-methyl-3-(6-(1-methyl-1*H*-3-imidazolium-3-yl)hexyl)-1*H*-imidazol-3-ium di(hydrogen sulfate) (**Cat1**) or 1-(6-(pyridinium-1-yl)hexyl)pyridin-1-ium di(hydrogen sulfate) (**Cat2**).

The two above-mentioned Brønsted-acidic ammonium hydrogen sulfate catalysts motivated us to study such multicomponent reactions using the ionic liquid (IL) catalysts 1-methyl-3-(6-(1-methyl-1*H*-3-imidazolium-3-yl)hexyl)-1*H*-imidazol-3-ium di(hydrogen sulfate) (Scheme 2, **Cat1**) [37,38,39] and 1-(6-(pyridinium-1-yl)hexyl)pyridin-1-ium di(hydrogen sulfate) (**Cat2**) [40] that have previously been used, especially for esterification reactions.

In continuation of our recent work using a heterogeneous sulfonic acid polyvinyl pyridinium IL catalyst for multi-component synthesis of spiroindole-3,5-pyrano[2,3-d]pyrimidines and pyrazines [41], we report herein the synthesis of spiro[furan-2,3′-indoline]-3-carboxylate derivatives using the two easy-to-make and versatile homogeneous Brønsted-acidic IL catalysts **Cat1** and **Cat2** in three-component reactions using isatin (*N*-alkyl-indoline-2,3-dione) derivatives, anilines, and diethyl acetylenedicarboxylate (Scheme 2). The use of ultrasonic irradiation was motivated by our own experience with such multi-component reactions suffering from limited miscibility of the components due to their different hydrophilic/hydrophobic properties and the wish to use only one solvent.

## 2. Results and Discussion

### 2.1. Optimization of the Reaction Conditions

**Scheme 3:**
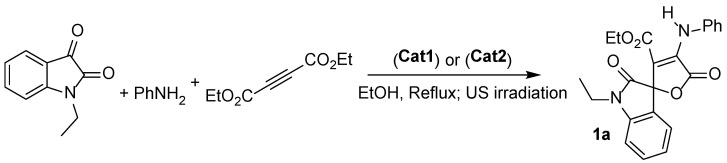
Synthesis of **1a** using **Cat1** = 1-methyl-3-(6-(1-methyl-1*H*-3-imidazolium-3-yl)hexyl)-1*H*-imidazol-3-ium di(hydrogen sulfate) or **Cat2** = 1-(6-(pyridinium-1-yl)hexyl)pyridin-1-ium di(hydrogen sulfate).

First, the synthesis of **1a** from the reaction of 1-ethylindoline-2,3-dione (1 mmol), aniline (1 mmol), and dimethylacetylenedicarboxylate (1 mmol) was studied under ultrasonic (US) irradiation, varying solvent, catalyst amounts, and temperature (Scheme 3). Without the catalyst and when the reaction was stirred at ambient temperature (r.t.) for 24 h, no product was obtained (Table 1). Similarly, when the reaction was heated at 80 °C under the solvent-free condition the substrates remain unreacted even after prolonged heating (24 h). Next the reaction was investigated under reflux conditions, and we found no product formation below 60 °C; among the solvents, EtOH was found to be suitable. Finally, we found that 30 mol% of the ionic liquid catalyst shows optimum performance with yields of 96% or 88% using **Cat1** or **Cat2**, respectively (Table 1).

Under the same conditions (EtOH, US, Reflux for 3 h), we also attempted the synthesis of **1a** using 30% acetic acid (HOAc) or sulfuric acid (H_2_SO_4_), but failed to detect the product in the reaction mixtures or after workup, although full conversion of the starting materials was achieved, as ^1^H NMR spectra unequivocally showed. In particular, the N-ethyl, O-ethyl, and the NH functions of the starting materials and product were indicative of this. Instead of the desired product, we obtained mixtures of unidentifiable products (Figures S31–S34, Supplementary Materials).

In a slightly different three-component [2+2+1] cycloaddition approach using diethyl acetylenedicarboxylate, *N*-substituted isatins and isocyanides, similar structures containing 4-alkyl- and aryl imine units, were previously obtained without using a catalyst in up to 89% yield after 18 h heating in toluene at 100 °C [42,43]. The use of 2,2-dimethyl-4,4-dimethyl-butane isocyanide and aqueous HCl workup allowed for the isolation of corresponding butenolides (C=O instead of imine) [42]. The reaction of PPh_3_ with diethyl acetylenedicarboxylate and *N*-alkylisatins was reported to give ethyl 2,2,2-triphenyl-2,5-dihydro-1,2-λ^5^-oxaphosphole-4-carboxylate-spiro-1-alkyl-1,3-dihydro-2*H*-indol-2-ones in 85% to 94% yields in 24 h at ambient *T* in CH_2_Cl_2_ [44]. This is fully in line with our failure to receive the product without the use of a catalyst and underlines our markedly superior catalyzed approach.

Other comparable [2+2+1] three-component reactions reported the use of aniline derivatives, dialkyl acetylenedicarboxylates and aromatic aldehydes to produce 3,4,5-substituted furan-2(5*H*)-one derivatives. Using (Bu_4_N)(HSO_4_) as homogeneous catalyst allowed reactions in EtOH at ambient *T* with up to 93% yields [35], which stands in contrast to our attempts to produce **1a** at ambient *T*, resulting in no product. However, the amount of catalyst used was not provided in this report. Elevated *T* of 45 °C in CH_2_Cl_2_ were necessary when using 2-pyrrolidonium hydrogen sulfate ((HNMP)(HSO_4_)) as catalyst (40 mol%) for similar products, which were obtained in up to 98% yield [36]. The best conditions for similar reactions using Al(HSO_4_)_3_ (31 mol%) as catalyst were EtOAc as solvent, 8 h at ambient T with 78% yields, whereas in EtOH the yield dropped to 51% [34]. A number of heterogeneous Lewis acidic catalysts have shown similar performance with maximum yields ranging from 75% to 97% and reaction times from 1 to 12 h, at elevated temperatures of up to 100 °C [26,27,28,29,30,31,32,33]. Within this frame, our catalyst systems perform on a good-to-excellent level.

### 2.2. Mechanistic Considerations

**Scheme 4:**
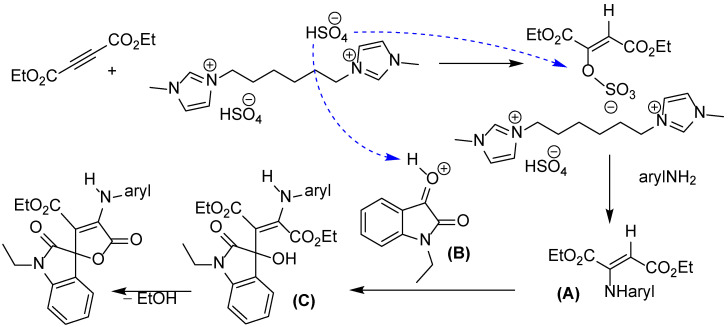
Probable mechanism for the ionic liquid catalyzed synthesis of 2′,5-dioxo-5*H*-spiro[furan-2,3′-indoline]-3-carboxylate derivatives. The dashed blue arrows illustrate the double role of HSO_4_^−^ attacking the triple bond and protonating the isatine C=O.

Mechanistically, the reaction is supposed to start via the sulfonation of the triple bond of the acetylenic ester and the subsequent replacement of the OSO_3_ group by the amine. Thus, diethyl 2-(phenylamino)fumarate is obtained as the intermediate (**A**). At the same time, 1-ethylindoline-2,3-dione is activated by ionic liquid through protonation at the 3-oxo group (**B**). The nucleophilic reaction of diethyl 2-(phenylamino)fumarate (**A**) with the protonated 1-ethylindoline-2,3-dione (**B**) gives the intermediate (**C**) which undergoes a cyclization reaction and elimination of EtOH (Scheme 4), while the catalyst is recycled.

For the three-component [2+2+1] cycloaddition reactions using diethyl acetylenedicarboxylate, *N*-substituted isatins, and 2,2-dimethyl-4,4-dimethyl-butane isonitrile, the attack of the isonitrile C atom to the triple bond in a Michael-type addition was proposed [42,43] and for the reaction of PPh_3_ with diethyl acetylenedicarboxylate and *N*-alkylisatins a dipolar intermediate resulting from the addition of the nucleophilic PPh_3_ to the triple bond was suggested [44]. In the following steps, a nucleophilic addition of the carbonyl (isatin) leads to intermediates being able to close the butyrolactone ring.

Importantly, both mechanisms are proposed for non-catalyzed reactions and their reaction mixtures both contain strong nucleophiles (isonitrile or PPh_3_). We have considered, alternatively to Scheme 4, that the isatin attacks the triple bond. However, in the above-mentioned mechanistic studies, this was not an option. Further, our failure to use the simple Brønstedt acids HOAc or H_2_SO_4_ as catalysts suggests that the IL-character of our catalysts, especially the ionic forces between the cationic pyridinium and imidazolium moieties and the anionic HSO_4_^−^, under the reaction conditions, make hydrogen sulfate a suitable nucleophile to attack the triple bond. However, the mechanistic details remain to be supported by studying the proposed sulfonated acetylenic esters and their reactivity towards amines, as well as by quantum chemical calculations. This will be the topic of a future study.

### 2.3. Studying the Substrate Scope

**Scheme 5:**
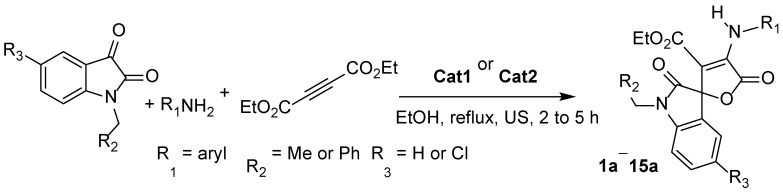
Syntheses of **9a**–**15a** using **Cat1** or **Cat2**.

Using the optimized conditions (EtOH, reflux, US, 3 h), the catalytic potential of ionic liquid catalysts on the preparation of further furan-2(5*H*)-ones was examined applying various anilines (Scheme 5). For products **1a**–**8a**, starting from 1-ethylindoline-2,3-dione, the yields ranged from 85% to 98% (Table 2). Relatively low yields and long reaction times were observed for 4-chloroaniline, which might be due to the electron-withdrawing nature of the chloride substituent. However, the reactions using 1-benzylindoline-2,3-dione (products **9a** to **11a**) or 5-chloroisatine (1-benzyl-5-chloro-indole-2,3-dione) (products **12a** to **15a**) did not show such a dependence. The same substrates were also studied using **Cat2**, and high yields were obtained (Table 2). Generally, the yields for **Cat2** lie slightly lower compared with the results for **Cat1**, which is in line with the results obtained already for the optimization of the synthesis of **1a** (Table 1). We ascribe this to the higher acidity of the HSO_4_^−^ moieties in **Cat1** compared with **Cat2**, which is in line with the protonation step in our proposed mechanism (Scheme 4).

In summary, the procedures proved to be effective in achieving a broad scope of 2′,5-dioxo-5*H*-spiro[furan-2,3′-indoline]-3-carboxylate derivatives in good-to-excellent yields. A simple filtration procedure allowed for the isolation of the pure products.

## 3. Conclusions

A simple synthetic method for the preparation of 2′,5-dioxo-5*H*-spiro[furan-2,3′-indoline]-3-carboxylate derivatives was described, which provides short reaction times and good yields using the ionic liquid catalysts 3,3′-(1,6-hexanediyl)bis(1-methyl)-1*H*-imidazolium hydrogen sulfate and 1,1′-(1,6-hexanediyl)bis(pyridinium) hydrogen sulfate. Importantly, the use of acidic acid (HOAc) or sulfuric acid (H_2_SO_4_) as catalysts under the same conditions failed. The IL catalysts are easy to prepare, cheap, can be easily separated, and showed high levels of catalytic activity for the three-component reaction of anilines, isatins (*N*-alkyl-indoline-2,3-diones), and diethyl acetylene dicarboxylate in EtOH under reflux, using ultrasonic (US) irradiation. The method is superior to non-catalyzed procedures, having far shorter reaction times and higher yields. Due to the complete conversion, only minute amounts of polymeric by-products were obtained, which can be separated together with the catalyst through centrifugation and filtration. Amongst other so far reported homogeneous acid catalysts, the separation of our systems is more convenient and does not require chromatographic separation of targeted products. Compared with heterogeneous acid catalysts, our systems are more reactive, and the simple handling makes our IL catalyst systems very interesting for further reactions catalyzed by Brønsted acids.

## 4. Materials and Methods

### 4.1. Reagents

1,6-dichlorohexane (Merck, Darmstadt, Germany, 98%), pyridine (Merck, ACS reagent, ≥99.0%), 1-methyl imidazole (Merck, Darmstadt, Germany, ReagentPLus, 99%), sulfuric acid (H_2_SO_4_, Merck, Darmstadt, Germany, ACS reagent 95.0-98.0%), diethyl acetylene dicarboxylate (Merck, 95%), 1-ethylindoline-2,3-dione (Merck, Darmstadt, Germany, Aldrich^CPR^, 99%), 1-benzyl-1*H*-indole-2,3-dione (Merck, Darmstadt, Germany, Aldrich^CPR^, 99%), 5-chloro-1-benzyl-1*H*-indole-2,3-dione (Biosynth AG, Staad, Switzerland), aniline (Merck, Darmstadt, Germany, ACS reagent, ≥99.5%), 4-methylaniline (toluidine, Merck, Darmstadt, Germany, 99%), 4-chloroaniline (Merck, Darmstadt, Germany, 98%), 4-methoxyaniline (anisidine, Merck, Darmstadt, Germany, 99%), 3,5-dimethylaniline (Fisher Scientific, Schwerte, Germany, 98%), 3,4-dimethylaniline (Fisher Scientific, 99.5%), 3,5-dimethoxyaniline (Fisher Scientific, Schwerte, Germany, 98%), 4-ethylaniline (Merck, Darmstadt, Germany, 98%) toluene (Fisher Scientific, 99%), MeOH (Merck, Darmstadt, Germany, for synthesis), EtOH (puriss. p.a., absolute, ≥99.8% (GC)), diethyl ether (Fisher Scientific, Schwerte, Germany, ExtraPure, BHT stabilized), and DMSO-d_6_ (Thermo Fisher, Schwerte, Germany, 99.5%) were commercially available and used without further purification. The catalysts **Cat1** and **Cat2** were synthesized from 1,6-dichlorohexane and *N*-methyl imidazole or pyridine, adopting reported methods (see Supplementary Materials) [38,39,40,41]. Analytic data were consistent with reported values.

### 4.2. Instrumentation

^1^H and ^13^C NMR spectra were recorded on a Bruker Avance DPX 500 MHz instrument (Bruker, Rheinhausen, Germany). Chemical shifts δ (in ppm) were given vs. the tetramethylsilane standard. A Heraeus CHN-O-Rapid analyzer (Heraeus, Darmstadt, Germany) was used for elemental analysis. Melting points were recorded on an Electrothermal IA9100 melting point apparatus (Electrothermal Ltd., London, UK) and are uncorrected. Thin-layer chromatography (TLC) was carried out using silica plates (Merck, Darmstadt, Germany) as the solid support and *n*-hexane/ethyl acetate (9/1) or c-hexane/ethyl acetate (2/1) as the eluting solvent.

### 4.3. General Procedure for the Syntheses

In a 50 mL flask equipped with a condenser, the ionic liquid catalyst (30 mol%) was added to a mixture of amine (1 mmol), diethyl acetylene dicarboxylate (1 mmol), and 1-ethylindoline-2,3-dione (1 mmol) in 20 mL EtOH under stirring. The mixture was irradiated using an ultrasonic bath at 80 °C. The progress of the reaction was monitored by thin-layer chromatography (TLC). After the reaction was completed, the solvent was evaporated to dryness and the residual solid was suspended in 5 mL EtOH and cooled. After centrifugation, the suspension was carefully poured through a 2 cm thick silica sludge deposited on a glass filter which collected the precipitated catalyst and polymeric by-products (yellow to brown sticky oil), while the pure products were obtained from the filtrate.

## Figures and Tables

**Table 1:** Optimization of the reaction conditions for the preparation of **1a ^a^**.

Conditions	Cat1	Cat2
	Time (h)/Yield (%)	Time (h)/Yield (%)
EtOH; US; Cat.: none; T (°C): r.t.	24/No product formed	24/No product formed
EtOH; US; Cat.: 30 mol%; T (°C): r.t.	24/No product formed	24/No product formed
EtOH; US; Cat.: 30 mol%; T (°C): Reflux	3/96	3/88
n-Hexane; US; Cat.: 30 mol%; T (°C): Reflux	24/No product formed	24/No product formed
CH_2_Cl_2_; US; Cat.: 30 mol%; T (°C): Reflux	24/No product formed	24/No product formed
Et_2_O; US; Cat.: 30 mol%; T (°C): Reflux	24/No product formed	24/No product formed
EtOAc; US; Cat.: 30 mol%; T (°C): Reflux	7/18	7/22
MeOH; US; Cat.: 30 mol%; T (°C): Reflux	4/66	4/61
H_2_O; US; Cat.: 30 mol%; T (°C): Reflux	5/31	5/45
Toluene; US; Cat.: 30 mol%; T (°C): Reflux	4/23	4/32
DMF; US; Cat.: 30 mol%; T (°C): Reflux	4/61	4/66
EtOH; US; Cat.: 5 mol%; T (°C): Reflux	3/26	3/18
EtOH; US; Cat.: 10 mol%; T (°C): Reflux	3/46	3/51
EtOH; US; Cat.: 20 mol%; T (°C): Reflux	3/76	3/79
EtOH; US; Cat.: 40 mol%; T (°C): Reflux	3/95	3/86
EtOH; US; Cat.: 50 mol%; T (°C): Reflux	3/94	3/87
EtOH; US; Cat.: 30 mol% H_2_SO_4_; T (°C): Reflux	3/No product formed ^b^	
EtOH; US; Cat.: 30 mol%; HOAc; T (°C): Reflux	3/No product formed ^b^	

^a^ Using 0.093 g aniline (1 mmol), 0.17 g diethyl acetylenedicarboxylate (1 mmol), and 0.175 g 1-ethylindoline-2,3-dione (1 mmol) in 20 mL EtOH. ^b^ Full conversion as monitored by ^1^H NMR and thin-layer chromatography (TLC).

**Table 2:** Synthesis of 2′,5-dioxo-5*H*-spiro[furan-2,3′-indoline]-3-carboxylate derivatives (**1a**–**15a**) ^a^.

Product	Substrate	Anilines	(Cat1)	(Cat2)	m.p. (°C)
	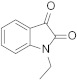		Time (h)/Yield (%) ^b^	Time (h)/Yield (%) ^b^	
**1a**		aniline	3/96	3/88	219–221
**2a**		4-methylaniline	2.5/98	2.5/85	223–225
**3a**		4-chloroaniline	5/85	5/80	245–247
**4a**		4-methoxyaniline	2.5/98	2.5/90	236–238
**5a**		3,5-dimethylaniline	2/97	2/92	249–251
**6a**		3,4-dimethylaniline	2/96	2/90	247–249
**7a**		3,5-dimethoxyaniline	2/93	2/96	255–257
**8a**		4-ethylaniline	2.5/94	2.5/93	231–233
	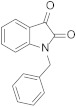				
**9a**		4-methylaniline	3.5/91	4/89	261–263
**10a**		4-chloroaniline	5/95	7/90	271–273
**11a**		4-methoxyaniline	3/96	3.5/91	277–279
	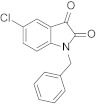				
**12a**		4-methylaniline	2.5/94	2.5/92	281–283
**13a**		4-chloroaniline	5/95	5/93	278–280
**14a**		4-methoxyaniline	3.5/91	3.5/91	272–274
**15a**		4-ethylaniline	4/96	4/93	275–277

^a^ Using 1 mmol aniline derivative, 1 mmol diethyl acetylene dicarboxylate (0.17 g), and 1 mmol isatine derivative in 20 mL EtOH, reflux, 2 to 5 h reaction time. ^b^ Isolated yields.

## Data Availability

Available from the authors on request.

## Supplementary Materials

The following supporting information can be downloaded at: https://www.mdpi.com/article/10.3390/molecules29061223/s1. Materials and Methods: Preparation of the ionic liquid catalyst **Cat1**. Scheme S1: Preparation of the ionic liquid catalyst **Cat1**. Preparation of the ionic liquid catalyst **Cat2**, Scheme S2: Preparation of the ionic liquid catalyst **Cat2**. Synthesis of ethyl 1′-R_2_-2′,5-dioxo-4-(R_1_-amino)-5H-spiro[5-R_3_-furan-2,3′-indoline]-3-carboxylates. Scheme S3: Synthesis of ethyl 1′-R_2_-2′,5-dioxo-4-(R_1_-amino)-5H-spiro[5-R_3_-furan-2,3′-indoline]-3-carboxylates. Figure S1: ^1^H NMR spectrum of **1a** in DMSO-d_6_. Figure S2: ^13^C NMR spectrum of **1a** in DMSO-d_6._ Figure S3: ^1^H NMR spectrum of **2a** in DMSO-d_6_. Figure S4: ^13^C NMR spectrum of **2a** in DMSO-d_6_. Figure S5: ^1^H NMR spectrum of **3a** in DMSO-d_6_. Figure S6: ^13^C NMR spectrum of **3a** in DMSO-d_6_. Figure S7: ^1^H NMR spectrum of **4a** in DMSO-d_6_ in DMSO-d_6_. Figure S8_:_ ^13^C NMR spectrum of **4a** in DMSO-d_6_. Figure S9: ^1^H NMR spectrum of 5a in DMSO-d_6_. Figure S10: ^13^C NMR spectrum of **5a** in DMSO-d_6_. Figure S11: ^1^H NMR spectrum of **6a** in DMSO-d_6_. Figure S12: ^13^C NMR spectrum of **6a** in DMSO-d_6_. Figure S13: ^1^H NMR spectrum of **7a** in DMSO-d_6_. Figure S14: ^13^C NMR spectrum of **7a** in DMSO-d_6_. Figure S15: ^1^H NMR spectrum of **8a** in DMSO-d_6_. Figure S16: ^13^C NMR spectrum of **8a** in DMSO-d_6_. Figure S17: ^1^H NMR spectrum of **9a** in DMSO-d_6_. Figure S18: ^13^C NMR spectrum of **9a** in DMSO-d_6_. Figure S19: ^1^H NMR spectrum of **10a** in DMSO-d_6_. Figure S20: ^13^C NMR spectrum of **10a** in DMSO-d_6_. Figure S21: ^1^H NMR spectrum of **11a** in DMSO-d_6_. Figure S22: ^13^C NMR spectrum of **11a** in DMSO-d_6_. Figure S23: ^1^H NMR spectrum of **12a** in DMSO-d_6_. Figure S24: ^13^C NMR spectrum of **12a** in DMSO-d_6_. Figure S25: ^1^H NMR spectrum of **13a** in DMSO-d_6_. Figure S26: ^13^C NMR spectrum of **13a** in DMSO-d_6_. Figure S27: ^1^H NMR spectrum of **14a** in DMSO-d_6_. Figure S28: ^13^C NMR spectrum of **14a** in DMSO-d_6_. Figure S29: ^1^H NMR spectrum of **15a** in DMSO-d_6_. Figure S30: ^13^C NMR spectrum of **15a** in DMSO-d_6_. Figure S31: Photograph of the TLC control of the reaction of aniline, diethyl acetylenedicarboxylate, 1-ethylindoline-2-,3-dione, using HOAc as catalyst. Figure S32: ^1^H NMR spectrum of the product mixture from the reaction of aniline, diethyl acetylenedicarboxylate, 1-ethylindoline-2-,3-dione, using HOAc as catalyst, in DMSO-d_6_. Figure S33: Photograph of the TLC control of the reaction of aniline, diethyl acetylenedicarboxylate, 1-ethylindoline-2-,3-dione, using H_2_SO_4_ as catalyst. Figure S34: ^1^H NMR spectrum of the product mixture from the reaction of aniline, diethyl acetylenedicarboxylate, 1-ethylindoline-2-,3-dione, using HOAc as catalyst, in DMSO-d_6_.
