# Supplementary material for: Multi-Component Syntheses of Spiro[furan-2,3′-indoline]-3-carboxylate Derivatives Using Ionic Liquid Catalysts
Source: Molecules. 2024 Mar 8;29(6):1223. doi: 10.3390/molecules29061223 (PMC10976190; doi:10.3390/molecules29061223)
Supplement: Supplementary file 1 [file molecules-29-01223-s001.zip › molecules-2874567-supplementary.pdf]

Supplementary Materials

Multi-Component Syntheses of Spiro[furan-2,3'-indoline]-3-carboxylate Derivatives Using Ionic Liquid Catalysts

Mehdi Khalaj [1,*], Maryam Zarandi [1], Malihe Samadi Kazemi [2], Seyed Mahmoud Musavi [1], Johannes Hohnsen [3], and Axel Klein [3,*]

1 Department of Chemistry, Buinzahra Branch, Islamic Azad University, Buinzahra 1477893855, Iran
2 Department of Chemistry, Faculty of Science, Bojnourd Branch, Islamic Azad University, Bojnourd 9417697796, Iran
3 Faculty of Mathematics and Natural Sciences, Department of Chemistry and Biochemistry, Institute for Inorganic Chemistry, University of Cologne, Greinstrasse 6, D-50939 Köln, Germany

* Correspondence: khalaj_mehdi@yahoo.com (M.K.); axel.klein@uni-koeln.de (A.K.)

Contents:

1. Materials and Methods

1.1 Preparation of the ionic liquid catalyst ***Cat1***
Scheme S1. Preparation of the ionic liquid catalyst **Cat1**
1.2 Preparation of the ionic liquid catalyst ***Cat2***
Scheme S2. Preparation of the ionic liquid catalyst **Cat2**
1.3 Synthesis of ethyl 1′-R_2-2′,5-dioxo-4-(R_1-amino)-5H-spiro[5-R_3-furan-2,3′-indoline]-3-carboxylates
Scheme S3. Synthesis of ethyl 1'-R_2-2',5-dioxo-4-(R_1-amino)-5*H*-spiro[5-R_3-furan-2,3'-indoline]-3-carboxylates

2. Supplementary Figures

Figure S1. ^{1}H NMR spectrum of **1a** in DMSO-d_6
Figure S2. ^{13}C NMR spectrum of **1a** in DMSO-d_6
Figure S3. ^{1}H NMR spectrum of **2a** in DMSO-d_6
Figure S4. ^{13}C NMR spectrum of **2a** in DMSO-d_6
Figure S5. ^{1}H NMR spectrum of **3a** in DMSO-d_6
Figure S6. ^{13}C NMR spectrum of **3a** in DMSO-d_6
Figure S7. ^{1}H NMR spectrum of **4a** in DMSO-d_6 in DMSO-d_6
Figure S8. ^{13}C NMR spectrum of **4a** in DMSO-d_6
Figure S9. ^{1}H NMR spectrum of **5a** in DMSO-d_6
Figure S10. ^{13}C NMR spectrum of **5a** in DMSO-d_6
Figure S11. ^{1}H NMR spectrum of **6a** in DMSO-d_6
Figure S12. ^{13}C NMR spectrum of **6a** in DMSO-d_6

Figure S13. ^{1}H NMR spectrum of **7a** in DMSO-d_6
Figure S14. ^{13}C NMR spectrum of **7a** in DMSO-d_6
Figure S15. ^{1}H NMR spectrum of **8a** in DMSO-d_6
Figure S16. ^{13}C NMR spectrum of **8a** in DMSO-d_6
Figure S17. ^{1}H NMR spectrum of **9a** in DMSO-d_6
Figure S18. ^{13}C NMR spectrum of **9a** in DMSO-d_6
Figure S19. ^{1}H NMR spectrum of **10a** in DMSO-d_6
Figure S20. ^{13}C NMR spectrum of **10a** in DMSO-d_6
Figure S21. ^{1}H NMR spectrum of **11a** in DMSO-d_6
Figure S22. ^{13}C NMR spectrum of **11a** in DMSO-d_6
Figure S23. ^{1}H NMR spectrum of **12a** in DMSO-d_6
Figure S24. ^{13}C NMR spectrum of **12a** in DMSO-d_6
Figure S25. ^{1}H NMR spectrum of **13a** in DMSO-d_6
Figure S26. ^{13}C NMR spectrum of **13a** in DMSO-d_6
Figure S27. ^{1}H NMR spectrum of **14a** in DMSO-d_6
Figure S28. ^{13}C NMR spectrum of **14a** in DMSO-d_6
Figure S29. ^{1}H NMR spectrum of **15a** in DMSO-d_6
Figure S30. ^{13}C NMR spectrum of **15a** in DMSO-d_6
Figure S31. Photograph of the TLC control of the reaction of aniline, diethyl acetylenedicarboxylate, 1-ethylindoline-2-,3-dione, using HOAc as catalyst.
Figure S32. ^{1}H NMR spectrum of the product mixture from the reaction of aniline, diethyl acetylenedicarboxylate, 1-ethylindoline-2-,3-dione, using HOAc as catalyst, measured in DMSO-d_6.
Figure S33. Photograph of the TLC control of the reaction of aniline, diethyl acetylenedicarboxylate, 1-ethylindoline-2-,3-dione, using H_2SO_4 as catalyst.
Figure S34. ^{1}H NMR spectrum of the product mixture from the reaction of aniline, diethyl acetylenedicarboxylate, 1-ethylindoline-2-,3-dione, using HOAc as catalyst, measured in DMSO-d_6.

1. Materials and Methods

*1.1 Preparation of the ionic liquid catalyst **Cat1***

8.211 g (10 mmol) *N*-methyl imidazole was mixed with 7.75 g (5 mmol) 1,6-dichlorohexane in 100 mL toluene and the mixture was heated under reflux overnight. The solvent was evaporated under vacuum. The thus obtained sample **(A1)** (3.18 g, 10 mmol, 99% yield) was dissolved in 100 mL MeOH, 1.96 g (20 mmol) sulfuric acid was added and the mixture stirred for 3 h. The reaction mixture was evaporated to dryness removing solvent and HCl from the mixture. The product was dried in vacuo (Scheme S1). Yield: 4.38 g (9.9 mmol, 99%) off-white powder. $C_{14}H_{26}N_4O_8S_2$ (442.50 g/mol). Elemental analysis calc. (found): C 38.00 (38.01), H 5.92 (5.89), 12.66 (12.67) %. ^{1}H NMR (300 MHz, DMSO-d_6) δ = 9.18 (s, 2H), 7.78 (m, 2H, *J* = 1.6 Hz), 7.72 (m, 2H), 4.15 (m, 4H, *J* = 7.2 Hz), 3.86 (s, 6H), 1.85–1.72 (m, 4H), 1.33–1.17 (m, 4H) ppm. No signals of residual 1,6-dichlorohexane or *N*-methyl imidazole were observed.

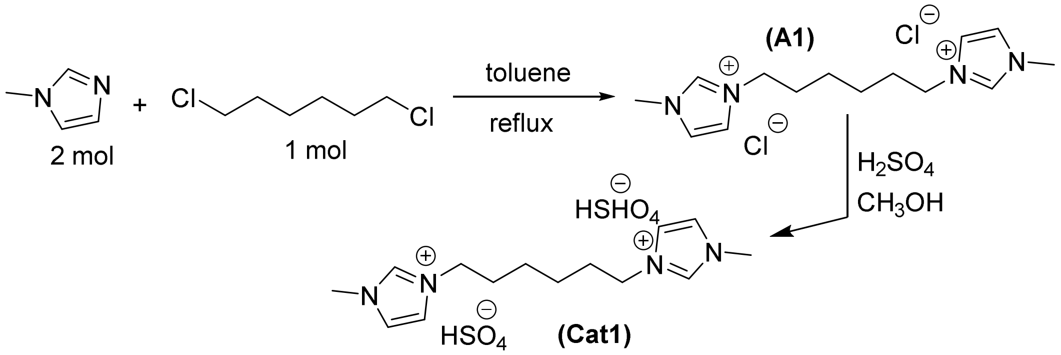

Scheme S1. Preparation of the ionic liquid catalyst **Cat1.**

*1.2 Preparation of the ionic liquid catalyst **Cat2***

7.91 g (10 mmol) pyridine was mixed with 7.75 g (5 mmol) 1,6-dichlorohexane in 100 mL toluene and the mixture was heated under reflux overnight. The solvent was evaporated under vacuum. The thus obtained sample **(A2)** (10 mmol, 3.13 g, 99% yield) was dissolved in 100 mL MeOH, 1.96 g (20 mmol) sulfuric acid was added and the mixture stirred for 3 h. The reaction mixture was evaporated to dryness removing solvent and HCl from the mixture. The product was dried in vacuo (Scheme S2). Yield: 4.32 g (9.9 mmol, 99%) off-white powder. $C_{16}H_{24}N_2O_8S_2$ (436.49 g/mol). Elemental analysis calc. (found): C 44.03 (44.10), H 5.54 (5.55), 6.42 (6.44) %. ^{1}H NMR (300 MHz, DMSO-d_6) δ = 9.13 (m, 4H), 8.65 (m, 2H, *J* = 7.7 Hz), 8.18 (m, 4H, *J* = 6.5 Hz), 4.74 (m, 4H, *J* = 7.5 Hz,), 2.12 (m, 4H), 1.57 (m, 4H) ppm. No signals of residual 1,6-dichlorohexane or pyridine were observed.

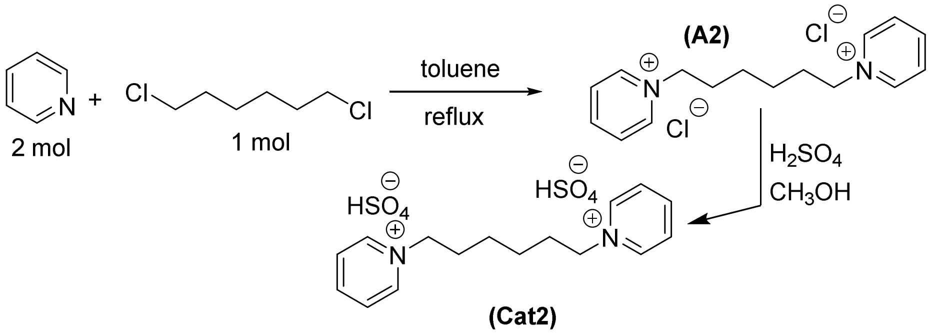

Scheme S2. Preparation of the ionic liquid catalyst **Cat2**

1.3 Synthesis of ethyl 1′-R2-2′,5-dioxo-4-(R1-amino)-5H-spiro[5-R3-furan-2,3′-indoline]-3-carboxylates – general optimized procedure

In a 50 mL flask equipped with a condenser, the ionic liquid catalyst **(Cat1)** (30 mol%) was added to a mixture of the aniline (1 mmol), diethyl acetylene dicarboxylate (0.17 g, 1 mmol), and 1-R2-indoline-2,3-dione (1 mmol) in 20 mL EtOH under stirring. The mixture was irradiated using an ultrasonic bath at 80 °C for a given period of time. The progress of the reaction was monitored by thin-layer chromatography (TLC). After completeness of the reaction, the solvent was evaporated to dryness and the residual solid was suspended in 5 mL EtOH and cooled. After centrifugation, the suspension was carefully poured through a 2 cm thick silica sludge deposited on a glass filter which collected the precipitated catalyst and polymeric by-products (yellow to brown sticky oil), while the pure products were obtained from the filtrate.

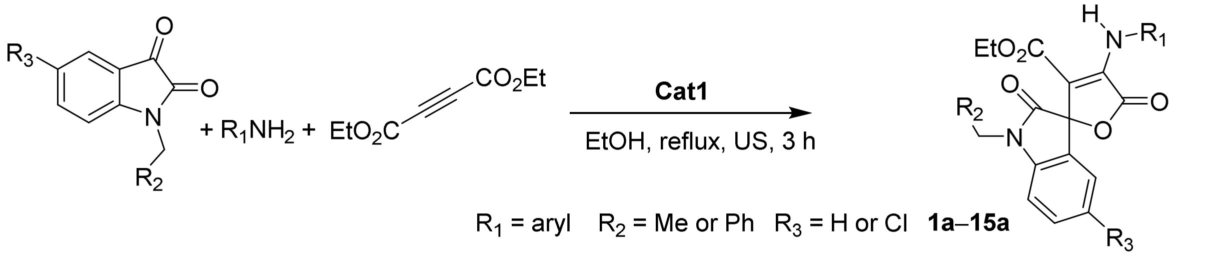

Scheme S3. Synthesis of ethyl 1'-R2-2',5-dioxo-4-(R1-amino)-5*H*-spiro[5-R3-furan-2,3'-indoline]-3-carboxylates

1a, Ethyl 1'-ethyl-2',5-dioxo-4-(phenylamino)-5*H*-spiro[furan-2,3'-indoline]-3-carboxylate
From 0.093 g aniline (1 mmol), 0.17 g diethyl acetylene dicarboxylate (1 mmol), and 0.175 g 1-ethylindoline-2,3-dione (1 mmol) in 20 mL EtOH for 3 h. Using **Cat1**: Yield 0.376 g (0.96 mmol, 96%), **Cat2**: Yield 0.345 g (0.88 mmol, 88%) colorless solid, m.p.: 219–221°C; ^{1}H NMR (500 MHz, DMSO-d$_6$): δ = 8.95 (s, 1H), 7.47 (t, *J* = 7.8 Hz, 1H), 7.31 (d, *J* = 7.8 Hz, 1H), 7.25 (t, *J* = 7.8 Hz, 2H), 7.16 (t, *J* = 7.8 Hz, 1H), 7.06–7.10 (m, 3H), 6.96 (d, *J* = 7.8 Hz, 1H), 4.11 (q, *J* = 6.8 Hz, 2H), 3.95 (q, *J* = 7.1 Hz, 2H), 1.24–1.28 (m, 6H) ppm; ^{13}C NMR (125 MHz, DMSO-d$_6$): δ = 14.2, 14.7, 48.7, 56.7, 89.4, 104.2, 114.1, 119.2, 120.3, 123.1, 124.5, 129.9, 134.5, 135.1, 143.8, 151.7, 155.1, 164.5, 169.3, 179.1 ppm; Found: C, 67.26; H, 5.02; N, 7.09% $C_{22}H_{20}N_2O_5$; requires: C, 67.34; H, 5.14; N, 7.14%.

2a, Ethyl 1'-ethyl-2',5-dioxo-4-(*p*-tolylamino)-5*H*-spiro[furan-2,3'-indoline]-3-carboxylate
From 0.107 g 4-methylaniline (1 mmol), 0.17 g diethyl acetylene dicarboxylate (1 mmol), and 0.175 g 1-ethylindoline-2,3-dione (1 mmol) in 20 mL EtOH for 2.5 h. Using **Cat1**: Yield 0.398 g (0.98 mmol, 98%), **Cat2**: Yield 0.345 g (0.85 mmol, 85%). Colorless solid, m.p.: 223–225°C; ^{1}H NMR (500 MHz, DMSO-d$_6$): δ = 8.56 (s, 1H), 7.48 (t, *J* = 7.9 Hz, 1H), 7.32 (d, *J* = 7.9 Hz, 1H), 7.14–7.19 (m, 3H), 6.97–7.01 (m, 3H), 4.13 (q, *J* = 7.0 Hz, 2H), 3.97 (q, *J* = 7.3 Hz, 2H), 2.29 (s, 3H), 1.22–1.29 (m, 6H) ppm; ^{13}C NMR (125 MHz, DMSO-d$_6$): δ = 14.3, 14.6, 21.4, 48.8, 56.4, 89.6, 104.1, 107.7, 112.3, 120.1, 124.6, 129.7, 134.1, 135.3, 137.1, 143.6, 151.9, 155.4, 164.8, 169.7, 178.7 ppm; Found: C, 67.87; H, 5.53; N, 6.83% $C_{23}H_{22}N_2O_5$; requires: C, 67.97; H, 5.46; N, 6.89%.

3a, Ethyl 4-((4-chlorophenyl)amino)-1'-ethyl-2',5-dioxo-5*H*-spiro[furan-2,3'-indoline]-3-carboxylate
From 0.127 g 4-chloroaniline (1 mmol), 0.17 g diethyl acetylene dicarboxylate (1 mmol), and 0.175 g 1-ethylindoline-2,3-dione (1 mmol) in 20 mL EtOH for 5 h. Using **Cat1**: Yield 0.362 g (0.85 mmol, 85%), **Cat2**: Yield 0.341 g (0.80 mmol, 80%) colorless solid, m.p.: 245–247°C; ^{1}H NMR (500 MHz, DMSO-d$_6$): δ = 9.32 (s, 1H), 7.49 (t, *J* = 8.2 Hz, 1H), 7.38 (d, *J* = 8.0 Hz, 2H), 7.32 (d, *J* = 8.2 Hz, 2H), 7.17 (t, *J* = 8.2 Hz, 1H), 7.09 (d, *J* = 8.0 Hz, 2H), 6.97 (d, *J* = 8.2 Hz, 1H), 4.13 (q, *J* = 7.0 Hz, 2H), 3.98 (q, *J* = 7.1 Hz, 2H), 1.21–1.29 (m, 6H) ppm; ^{13}C NMR (125 MHz, DMSO-d$_6$): δ = 14.4, 15.1, 49.2, 56.5, 91.2, 106.3, 118.7, 120.3, 125.2, 127.9, 129.8, 134.4,

136.1, 143.7, 144.2, 152.3, 156.1, 165.7, 170.1, 179.4 ppm; Found: C, 61.86; H, 4.55; N, 6.63% $C_{22}H_{19}ClN_2O_5$; requires: C, 61.90; H, 4.49; N, 6.56%.

4a, Ethyl 1'-ethyl-2',5-dioxo-4-((4-methoxyphenyl)amino)-5*H*-spiro[furan-2,3'-indoline]-3-carboxylate
From 0.123 g 4-methoxyaniline (1 mmol), 0.17 g diethyl acetylene dicarboxylate (1 mmol), and 0.175 g 1-ethylindoline-2,3-dione (1 mmol) in 20 mL EtOH for 2.5 h. Using **Cat1**: Yield 0.414 g (0.98 mmol, 98%), **Cat2**: Yield 0.38 g (0.90 mmol, 90%) colorless solid, m.p.: 236–238°C; ^{1}H NMR (500 MHz, DMSO-d$_6$): δ = 9.12 (s, 1H), 7.47 (t, *J* = 8.0 Hz, 1H), 7.31 (d, *J* = 8.0 Hz, 1H), 7.16 (t, *J* = 8.0 Hz, 1H), 7.03 (d, *J* = 8.1 Hz, 2H), 6.96 (d, *J* = 8.0 Hz, 1H), 6.87 (d, *J* = 8.1 Hz, 2H), 4.09 (q, *J* = 6.9 Hz, 2H), 3.96 (q, *J* = 6.8 Hz, 2H), 3.74 (s, 3H), 1.20–1.29 (m, 6H) ppm; ^{13}C NMR (125 MHz, DMSO-d$_6$): δ = 14.4, 14.9, 48.3, 55.4, 56.3, 89.2, 103.2, 106.2, 110.3, 119.8, 124.1, 129.9, 134.4, 134.8, 143.2, 151.1, 155.1, 155.7, 164.1, 167.3, 176.1 ppm; Found: C, 65.33; H, 5.26; N, 6.58% $C_{23}H_{22}N_2O_6$; requires: C, 65.40; H, 5.25; N, 6.63%.

5a, Ethyl 4-((3,5-dimethylphenyl)amino)-1'-ethyl-2',5-dioxo-5*H*-spiro[furan-2,3'-indoline]-3-carboxylate
From 0.121 g 3,5-dimethylaniline (1 mmol), 0.17 g diethyl acetylene dicarboxylate (1 mmol), and 0.175 g 1-ethylindoline-2,3-dione (1 mmol) in 20 mL EtOH for 2 h. Using **Cat1**: Yield 0.407 g (0.97 mmol, 97%), **Cat2**: Yield 0.386 g (0.92 mmol, 92%) colorless solid, m.p.: 249–251°C; ^{1}H NMR (500 MHz, DMSO-d$_6$): δ = 8.41 (s, 1H), 7.46 (t, *J* = 8.0 Hz, 1H), 7.30 (d, *J* = 8.0 Hz, 1H), 7.15 (t, *J* = 8.0 Hz, 1H), 6.95 (d, *J* = 8.0 Hz, 1H), 6.88 (s, 1H), 6.85 (s, 2H), 4.03 (q, *J* = 7.0 Hz, 2H), 3.92 (q, *J* = 7.1 Hz, 2H), 2.27 (s, 6H), 1.20–1.27 (m, 6H) ppm; ^{13}C NMR (125 MHz, DMSO-d$_6$): δ = 14.3, 15.0, 21.6, 48.6, 56.5, 89.0, 105.2, 111.2, 116.7, 119.7, 124.2, 129.7, 134.1, 135.8, 137.3, 143.1, 151.0, 155.5, 163.8, 166.0, 178.1 ppm; Found: C, 68.45; H, 5.66; N, 6.58% $C_{24}H_{24}N_2O_5$; requires: C, 68.56; H, 5.75; N, 6.66%.

6a, Ethyl 4-((3,4-dimethylphenyl)amino)-1'-ethyl-2',5-dioxo-5*H*-spiro[furan-2,3'-indoline]-3-carboxylate
From 0.121 g 3,4-dimethylaniline (1 mmol), 0.17 g diethyl acetylene dicarboxylate (1 mmol), and 0.175 g 1-ethylindoline-2,3-dione (1 mmol) in 20 mL EtOH for 2 h. Using **Cat1**: Yield 0.403 g (0.96 mmol, 96%), **Cat2**: Yield 0.378 g (0.90 mmol, 90%) colorless solid, m.p.: 247–249°C; ^{1}H NMR (500 MHz, DMSO-d$_6$): δ = 7.89 (s, 1H), 7.46 (t, *J* = 8.2 Hz, 1H), 7.31 (d, *J* = 8.2 Hz, 1H), 7.15 (t, *J* = 8.2 Hz, 1H), 7.07 (d, *J* = 7.8 Hz, 1H), 7.04 (d, *J* = 7.8 Hz, 1H), 6.98 (s, 1H), 6.95 (d, *J* = 8.2 Hz, 1H), 4.04 (q, *J* = 7.0 Hz, 2H), 3.93 (q, *J* = 7.0 Hz, 2H), 2.78 (s, 3H), 2.26 (s, 3H), 1.27 (t, *J* = 7.0 Hz, 3H), 1.22 (t, *J* = 7.0 Hz, 3H) ppm; ^{13}C NMR (125 MHz, DMSO-d$_6$): δ = 14.3, 14.9, 20.8, 21.3, 48.7, 56.5, 89.4, 105.7, 109.9, 112.4, 116.1, 119.8, 124.5, 129.8, 134.2, 135.4, 136.8, 137.1, 143.3, 151.1, 155.6, 163.7, 166.2, 177.7 ppm; Found: C, 68.57; H, 5.69; N, 6.65% $C_{24}H_{24}N_2O_5$; requires: C, 68.56; H, 5.75; N, 6.66%.

7a, Ethyl 4-((3,5-dimethoxyphenyl)amino)-1'-ethyl-2',5-dioxo-5*H*-spiro[furan-2,3'-indoline]-3-carboxylate
From 0.153 g 3,5-dimethoxyaniline (1 mmol), 0.17 g diethyl acetylene dicarboxylate (1 mmol), and 0.175 g 1-ethylindoline-2,3-dione (1 mmol) in 20 mL EtOH for 2 h. Using **Cat1**: Yield 0.420 g (0.93 mmol, 93%), **Cat2**: Yield 0.434 g (0.96 mmol, 96%) colorless solid, m.p.: 255–257°C; ^{1}H NMR (500 MHz, DMSO-d$_6$): δ = 8.36 (s, 1H), 7.47 (t, *J* = 8.1 Hz, 1H), 7.31 (d, *J* = 8.1 Hz, 1H), 7.16 (t, *J* = 8.1 Hz, 1H), 6.96 (d, *J* = 8.1 Hz, 1H), 6.81 (s, 1H), 6.73 (s, 2H), 4.08 (q, *J* = 7.0 Hz, 2H), 3.95 (q, *J* = 6.9 Hz, 2H), 3.71 (s, 6H), 1.20-1.28 (m, 6H) ppm; ^{13}C NMR (125 MHz, DMSO-d$_6$): δ = 14.1, 15.0, 48.6, 55.6, 56.7, 88.7, 104.2, 105.8, 109.7, 119.8, 124.4, 129.7, 134.0, 135.6, 143.2, 150.7, 155.2, 155.8, 163.1, 165.2, 174.1 ppm; Found: C, 63.73; H, 5.37; N, 6.19% $C_{24}H_{24}N_2O_7$; requires: C, 63.71; H, 5.35; N, 6.19%.

8a, Ethyl 1'-ethyl-4-((4-ethylphenyl)amino)-2',5-dioxo-5H-spiro[furan-2,3'-indoline]-3-carboxylate
From 0.121 g 4-ethylaniline (1 mmol), 0.17 g diethyl acetylene dicarboxylate (1 mmol), and 0.175 g 1-ethylindoline-2,3-dione (1 mmol) in 20 mL EtOH for 2.5 h. Using **Cat1**: Yield 0.395 g (0.94 mmol, 94%), **Cat2**: Yield 0.391 g (0.93 mmol, 93%). Colorless solid, m.p.: 231–233°C; ^{1}H NMR (500 MHz, DMSO-d$_6$): δ = 7.96 (s,

1H), 7.47 (t, *J* = 8.0 Hz, 1H), 7.32 (d, *J* = 8.0 Hz, 1H), 7.16 (t, *J* = 8.0 Hz, 1H), 7.09 (d, *J* = 7.8 Hz, 2H), 7.01 (d, *J* = 7.8 Hz, 2H), 6.96 (d, *J* = 8.0 Hz, 1H), 4.08 (q, *J* = 6.8 Hz, 2H), 3.96 (q, *J* = 6.8 Hz, 2H), 2.19 (q, *J* = 7.0 Hz, 2H), 1.22–1.30 (m, 6H), 1.04 (t, *J* = 7.0 Hz, 3H) ppm; ^{13}C NMR (125 MHz, DMSO-d$_6$): δ = 13.9, 14.4, 15.0, 24.8, 48.6, 56.5, 89.1, 104.5, 111.1, 115.3, 119.9, 124.5, 129.8, 134.2, 135.4, 137.7, 143.4, 151.2, 155.6, 164.2, 168.7, 177.1 ppm; Found: C, 68.66; H, 5.89; N, 6.74% $C_{24}H_{24}N_2O_5$; requires: C, 68.56; H, 5.75; N, 6.66%.

9a, Ethyl 1'-benzyl-2',5-dioxo-4-(*p*-tolylamino)-5*H*-spiro[furan-2,3'-indoline]-3-carboxylate **(Table 2, 9a):** From 0.107 g 4-methylaniline (1 mmol), 0.17 g diethyl acetylene dicarboxylate (1 mmol), and 0.237 g 1-bezylindoline-2,3-dione (1 mmol) in 20 mL EtOH for 3.5 h. Using **Cat1**: Yield 0.426 g (0.91 mmol, 91%), **Cat2**: Yield 0.417 g (0.89 mmol, 89%) colorless solid, m.p.: 261–263°C; ^{1}H NMR (500 MHz, DMSO-d$_6$): δ = 7.76 (s, 1H), 7.49 (t, *J* = 8.0 Hz, 1H), 7.34 (d, *J* = 8.0 Hz, 1H), 7.13–7.25 (m, 6H), 7.03 (d, *J* = 8.0 Hz, 2H), 6.97 (d, *J* = 8.0 Hz, 1H), 6.91 (d, *J* = 8.0 Hz, 2H), 4.58 (d, *J* = 10.8 Hz, 1H), 4.48 (d, *J* = 10.8 Hz, 1H), 4.16 (q, *J* = 6.8 Hz, 2H), 2.28 (s, 3H), 1.31 (t, *J* = 6.8 Hz, 3H) ppm; ^{13}C NMR (125 MHz, DMSO-d$_6$): δ = 15.8, 21.4, 56.7, 74.6, 91.7, 105.7, 112.2, 116.3, 121.2, 124.7, 127.6, 128.8, 129.1, 129.8, 134.6, 136.7, 137.8, 138.6, 143.2, 151.5, 155.1, 167.2, 169.7, 177.7 ppm; Found: C, 71.85; H, 5.22; N, 5.91% $C_{28}H_{24}N_2O_5$; requires: C, 71.78; H, 5.16; N, 5.98%.

10a, Ethyl 1'-benzyl-4-((4-chlorophenyl)amino)-2',5-dioxo-5H-spiro[furan-2,3'-indoline]-3-carboxylate From 0.127 g 4-chloroaniline (1 mmol), 0.17 g diethyl acetylene dicarboxylate (1 mmol), and 0.237 g 1-bezylindoline-2,3-dione (1 mmol) in 20 mL EtOH for 5 h. Using **Cat1**: Yield 0.464 g (0.95 mmol, 95%), **Cat2**: Yield 0.439 g (0.90 mmol, 90%) colorless solid, m.p.: 271–273°C; ^{1}H NMR (500 MHz, DMSO-d$_6$): δ = 8.87 (s, 1H), 7.50 (t, *J* = 8.2 Hz, 1H), 7.41 (d, *J* = 7.8 Hz, 2H), 7.35 (d, *J* = 8.2 Hz, 1H), 7.14–7.26 (m, 6H), 7.08 (d, *J* = 7.8 Hz, 2H), 6.96 (d, *J* = 8.2 Hz, 1H), 4.60 (d, *J* = 10.9 Hz, 1H), 4.51 (d, *J* = 10.8 Hz, 1H), 4.14 (q, *J* = 6.8 Hz, 2H), 1.30 (t, *J* = 6.9 Hz, 3H) ppm; ^{13}C NMR (125 MHz, DMSO-d$_6$): δ = 16.1, 57.1, 74.9, 92.3, 106.1, 121.3, 124.8, 127.2, 127.8, 128.4, 129.0, 129.4, 130.1, 134.7, 136.6, 138.7, 143.6, 144.2, 151.4, 155.3, 167.7, 170.1, 178.4 ppm; Found: C, 66.42; H, 4.39; N, 5.67% $C_{27}H_{21}ClN_2O_5$; requires: C, 66.33; H, 4.33; N, 5.73%.

11a, Ethyl 1'-benzyl-4-((4-methoxyphenyl)amino)-2',5-dioxo-5*H*-spiro[furan-2,3'-indoline]-3-carboxylate From 0.123 g 4-methoxyaniline (1 mmol), 0.17 g diethyl acetylene dicarboxylate (1 mmol), and 0.237 g 1-bezylindoline-2,3-dione (1 mmol) in 20 mL EtOH for 3h. Using **Cat1**: Yield 0.465 g (0.96 mmol, 96%), **Cat2**: Yield 0.441 g (0.91 mmol, 91%) colorless solid, m.p.: 277–279°C; ^{1}H NMR (500 MHz, DMSO-d$_6$): δ = 8.47 (s, 1H), 7.49 (t, *J* = 8.2 Hz, 1H), 7.35 (d, *J* = 8.1 Hz, 1H), 7.25 (d, *J* = 7.8 Hz, 2H), 7.13–7.21 (m, 4H), 6.96 (d, *J* = 8.1 Hz, 1H), 6.91 (d, *J* = 7.8 Hz, 2H), 6.81 (d, *J* = 7.8 Hz, 2H), 4.60 (d, *J* = 11.0 Hz, 1H), 4.50 (d, *J* = 11.0 Hz, 1H), 4.11 (q, *J* = 6.8 Hz, 2H), 3.69 (s, 3H), 1.30 (t, *J* = 6.8 Hz, 3H) ppm; ^{13}C NMR (125 MHz, DMSO-d$_6$): δ = 16.0, 55.2, 56.8, 74.3, 92.0, 105.8, 107.3, 110.8, 121.0, 124.4, 127.4, 128.2, 129.2, 130.0, 134.3, 135.1, 138.5, 143.4, 151.2, 155.4, 155.7, 166.3, 169.1, 175.2 ppm; Found: C, 69.49; H, 5.01; N, 5.78% $C_{28}H_{24}N_2O_6$; requires: C, 69.41; H, 4.99; N, 5.78%.

12a, Ethyl 1'-benzyl-5'-chloro-2',5-dioxo-4-(p-tolylamino)-5*H*-spiro[furan-2,3'-indoline]-3-carboxylate From 0.107 g 4-methylaniline (1 mmol), 0.17 g diethyl acetylene dicarboxylate (1 mmol), and 0.271 g 1-benzyl-5-chloroindoline-2,3-dione (1 mmol) in 20 mL EtOH for 2.5 h. Using **Cat1**: Yield 0.472 g (0.94 mmol, 94%), **Cat2**: Yield 0.462 g (0.92 mmol, 92%) colorless solid, m.p.: 281–283°C; ^{1}H NMR (500 MHz, DMSO-d$_6$): δ = 8.87 (s, 1H), 6.93–7.29 (m, 12H), 4.62 (d, *J* = 11.0 Hz, 1H), 4.53 (d, *J* = 11.0 Hz, 1H), 4.10 (q, *J* = 6.8 Hz, 2H), 2.25 (s, 3H), 1.28 (t, *J* = 6.8 Hz, 3H) ppm; ^{13}C NMR (125 MHz, DMSO-d$_6$): δ = 16.3, 21.2, 56.7, 74.7, 93.1, 106.8, 112.3, 116.8, 122.3, 126.7, 127.4, 128.4, 129.3, 132.3, 135.3, 136.4, 137.5, 143.6, 144.6, 151.5, 155.6, 166.7, 170.1, 176.4 ppm; Found: C, 66.87; H, 4.60; N, 5.59% $C_{28}H_{23}ClN_2O_5$; requires: C, 66.87; H, 4.61; N, 5.57%.

13a, Ethyl 1'-benzyl-5'-chloro-4-((4-chlorophenyl)amino)-2',5-dioxo-5H-spiro[furan-2,3'-indoline]-3-carboxylate

From 0.127 g 4-chloroaniline (1 mmol), 0.17 g diethyl acetylene dicarboxylate (1 mmol), and 0.271 g 1-benzyl-5-chloroindoline-2,3-dione (1 mmol) in 20 mL EtOH for 5 h. Using **Cat1**: Yield 0.496 g (0.95 mmol, 95%), **Cat2**: Yield 0.486 g (0.93 mmol, 93%) colorless solid, m.p.: 278–280°C; ^{1}H NMR (500 MHz, DMSO-d$_6$): δ = 8.96 (s, 1H), 7.41 (d, *J* = 7.8 Hz, 2H), 7.14–7.30 (m, 7H), 7.09 (d, *J* = 7.8 Hz, 2H), 6.96 (d, *J* = 8.0 Hz, 1H), 4.61 (d, *J* = 10.6 Hz, 1H), 4.51 (d, *J* = 10.6 Hz, 1H), 4.11 (q, *J* = 6.8 Hz, 2H), 1.29 (t, *J* = 6.9 Hz, 3H) ppm; ^{13}C NMR (125 MHz, DMSO-d$_6$): δ = 16.4, 56.6, 76.1, 94.2, 106.7, 122.1, 126.4, 127.1, 127.6, 128.1, 128.5, 129.4, 132.1, 135.4, 136.8, 143.2, 143.8, 144.5, 151.6, 155.7, 166.5, 170.6, 178.4 ppm; Found: C, 61.84; H, 3.77; N, 5.26% $C_{27}H_{20}Cl_2N_2O_5$; requires: C, 61.96; H, 3.85; N, 5.35%.

14a, Ethyl 1'-benzyl-5'-chloro-4-((4-methoxyphenyl)amino)-2',5-dioxo-5H-spiro[furan-2,3'-indoline]-3-carboxylate

From 0.123 g 4-methoxyaniline (1 mmol), 0.17 g diethyl acetylene dicarboxylate (1 mmol), and 0.271 g 1-benzyl-5-chloroindoline-2,3-dione (1 mmol) in 20 mL EtOH for 3.5 h. Using **Cat1**: Yield 0.472 g (0.91 mmol, 91%), **Cat2**: Yield 0.471 g (0.91 mmol, 91%) colorless solid, m.p.: 272–274°C; ^{1}H NMR (500 MHz, DMSO-d$_6$): δ = 8.56 (s, 1H), 7.13–7.29 (m, 7H), 6.96 (d, *J* = 8.2 Hz, 1H), 6.89 (d, *J* = 7.8 Hz, 2H), 6.77 (d, *J* = 7.9 Hz, 2H), 4.59 (d, *J* = 10.8 Hz, 1H), 4.49 (d, *J* = 10.8 Hz, 1H), 4.09 (q, *J* = 6.9 Hz, 2H), 3.89 (s, 3H), 1.28 (t, *J* = 6.9 Hz, 3H) ppm; ^{13}C NMR (125 MHz, DMSO-d$_6$): δ = 15.8, 55.4, 56.1, 75.2, 93.8, 106.1, 108.4, 112.3, 121.7, 126.0, 127.3, 128.4, 129.2, 132.0, 135.1, 135.4, 143.1, 144.3, 151.2, 155.3, 155.5, 165.2, 169.6, 177.1 ppm; Found: C, 64.75; H, 4.41; N, 5.33% $C_{28}H_{23}ClN_2O_6$; requires: C, 64.81; H, 4.47; N, 5.40%.

15a, Ethyl 1'-benzyl-5'-chloro-4-((4-ethylphenyl)amino)-2',5-dioxo-5H-spiro[furan-2,3'-indoline]-3-carboxylate

From 0.121 g 4-ethylaniline (1 mmol), 0.17 g diethyl acetylene dicarboxylate (1 mmol), and 0.271 g 1-benzyl-5-chloroindoline-2,3-dione (1 mmol) in 20 mL EtOH for 4 h. Using **Cat1**: Yield 0.496 g (0.96 mmol, 96%), **Cat2**: Yield 0.480 g (0.93 mmol, 93%) colorless solid, m.p.: 275–277°C; ^{1}H NMR (500 MHz, DMSO-d$_6$): δ = 8.36 (s, 3H), 6.95–7.30 (m, 12H), 4.59 (d, *J* = 10.8 Hz, 1H), 4.46 (d, *J* = 10.8 Hz, 1H), 4.08 (q, *J* = 6.8 Hz, 2H), 2.35 (q, *J* = 6.8 Hz, 2H), 1.29 (t, *J* = 6.8 Hz, 3H), 0.98 (t, *J* = 6.8 Hz, 3H) ppm; ^{13}C NMR (125 MHz, DMSO-d$_6$): δ = 14.6, 15.7, 24.3, 56.1, 75.1, 93.5, 106.1, 112.4, 117.3, 121.8, 125.9, 127.3, 128.4, 129.2, 132.1, 135.3, 135.8, 136.8, 143.1, 144.4, 151.2, 155.5, 165.6, 169.1, 177.4 ppm; Found: C, 67.43; H, 4.96; N, 5.48% $C_{29}H_{25}ClN_2O_5$; requires: C, 67.38; H, 4.87; N, 5.42%.

2. Supplementary Figures

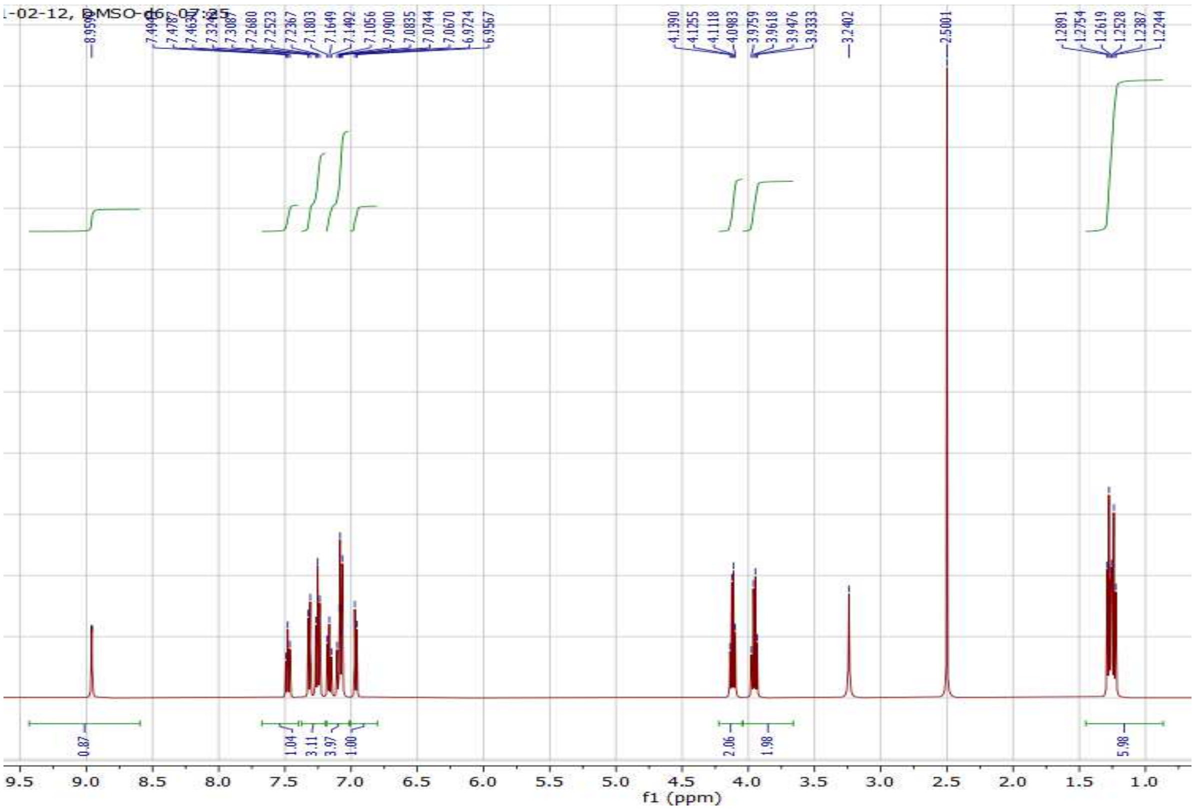

Figure S1. ^{1}H NMR spectrum of **1a** in DMSO-d_6

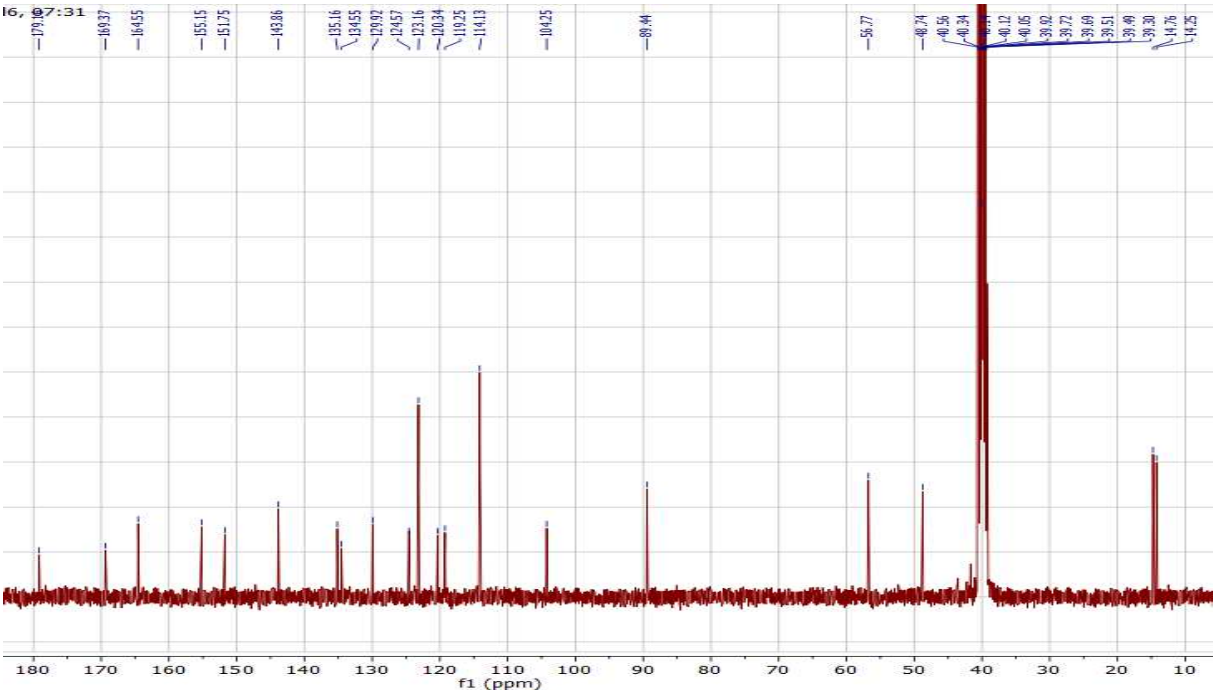

Figure S2. ^{13}C NMR spectrum of **1a** in DMSO-d_6

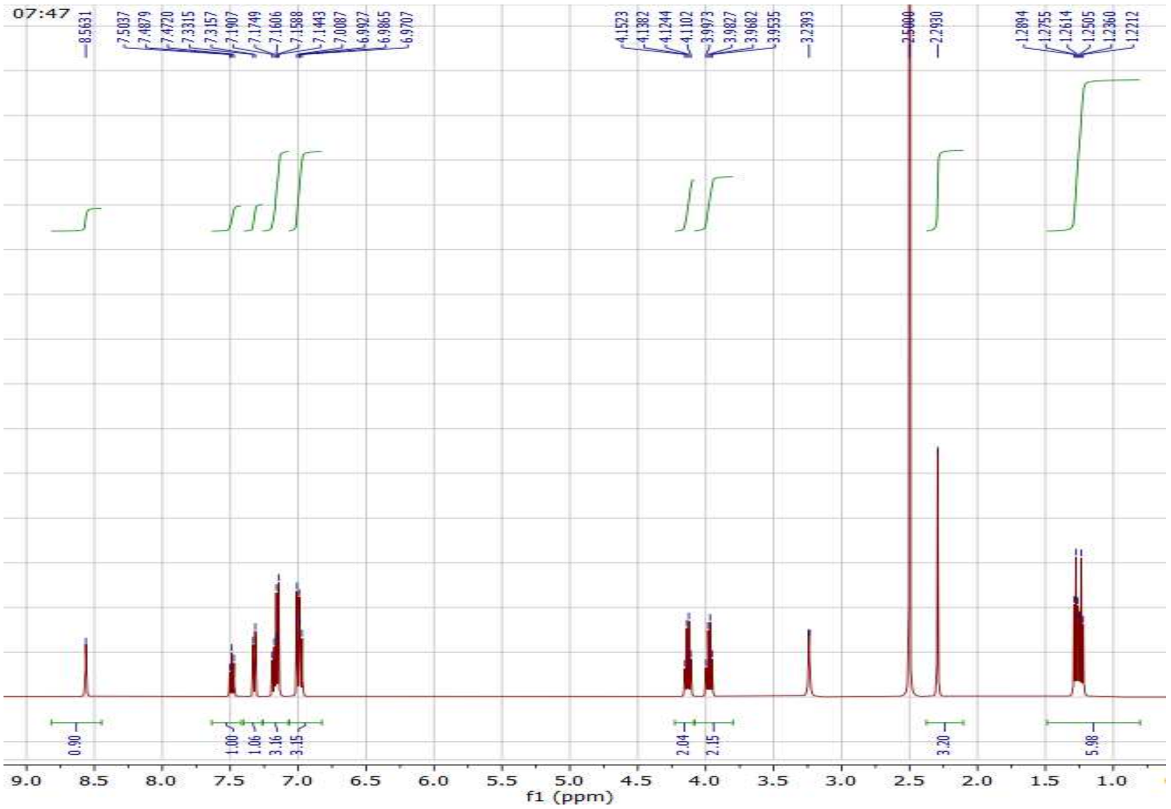

Figure S3. ^{1}H NMR spectrum of **2a** in DMSO-d_6

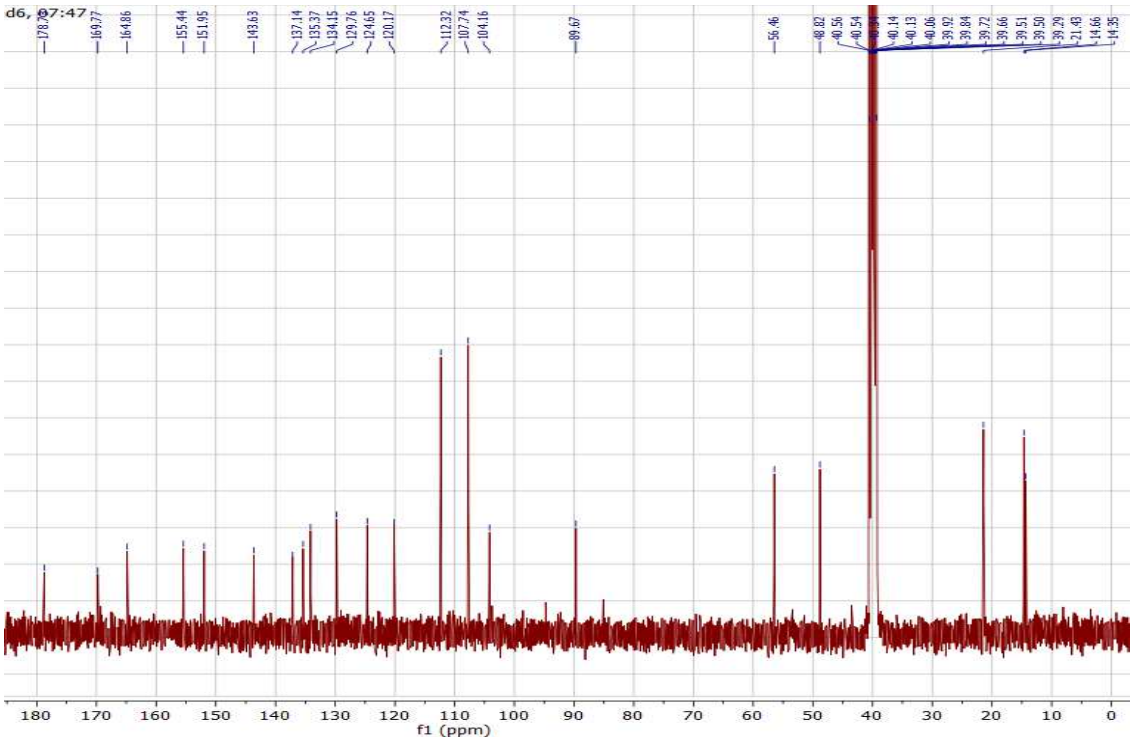

Figure S4. ^{13}C NMR spectrum of **2a** in DMSO-d_6

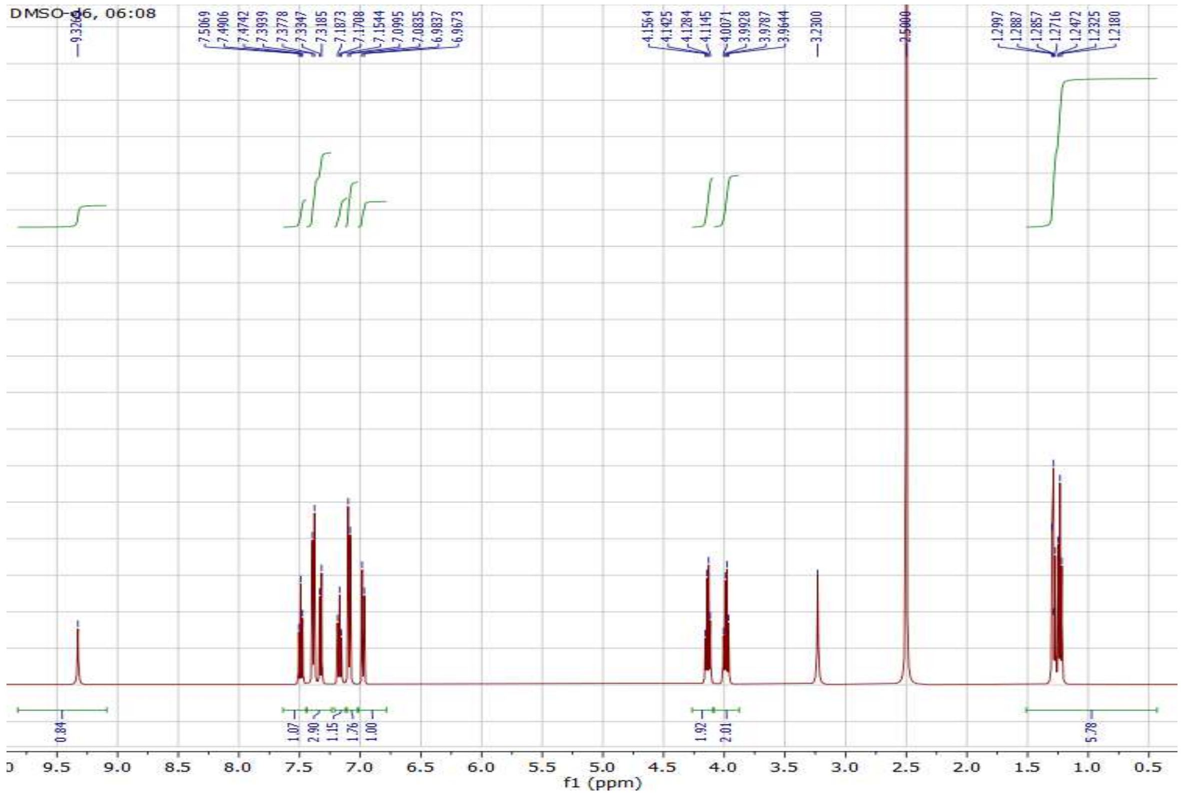

Figure S5. ^{1}H NMR spectrum of **3a** in DMSO-d_6

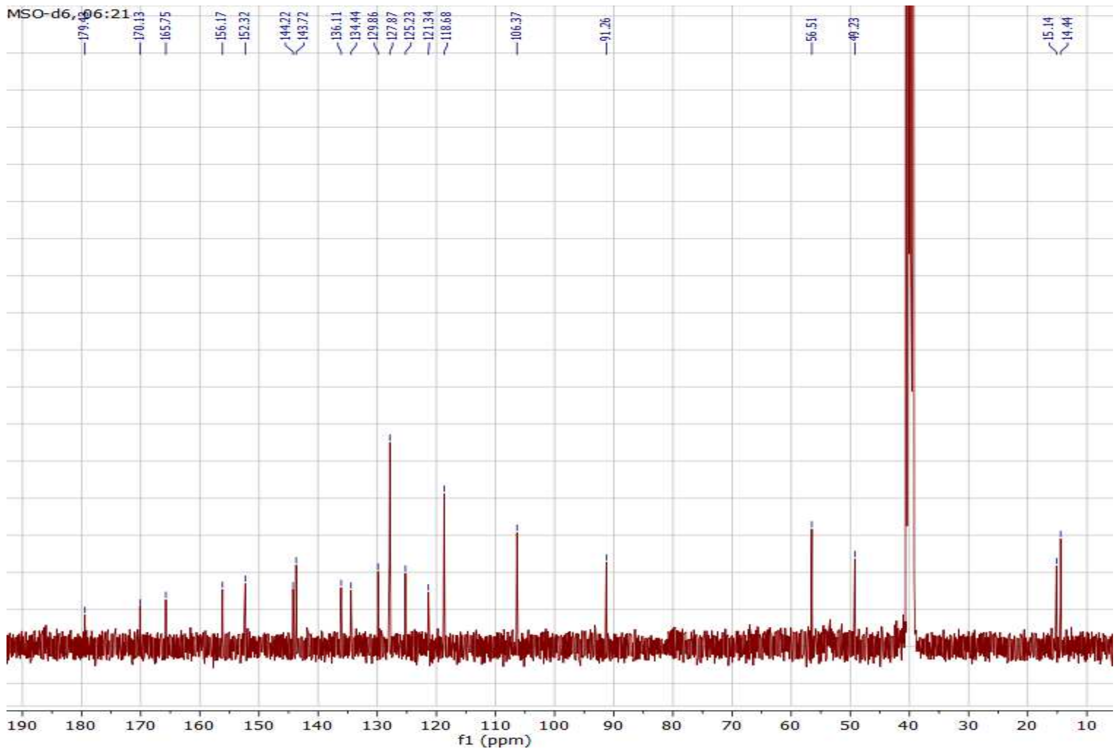

Figure S6. ^{13}C NMR spectrum of **3a** in DMSO-d_6

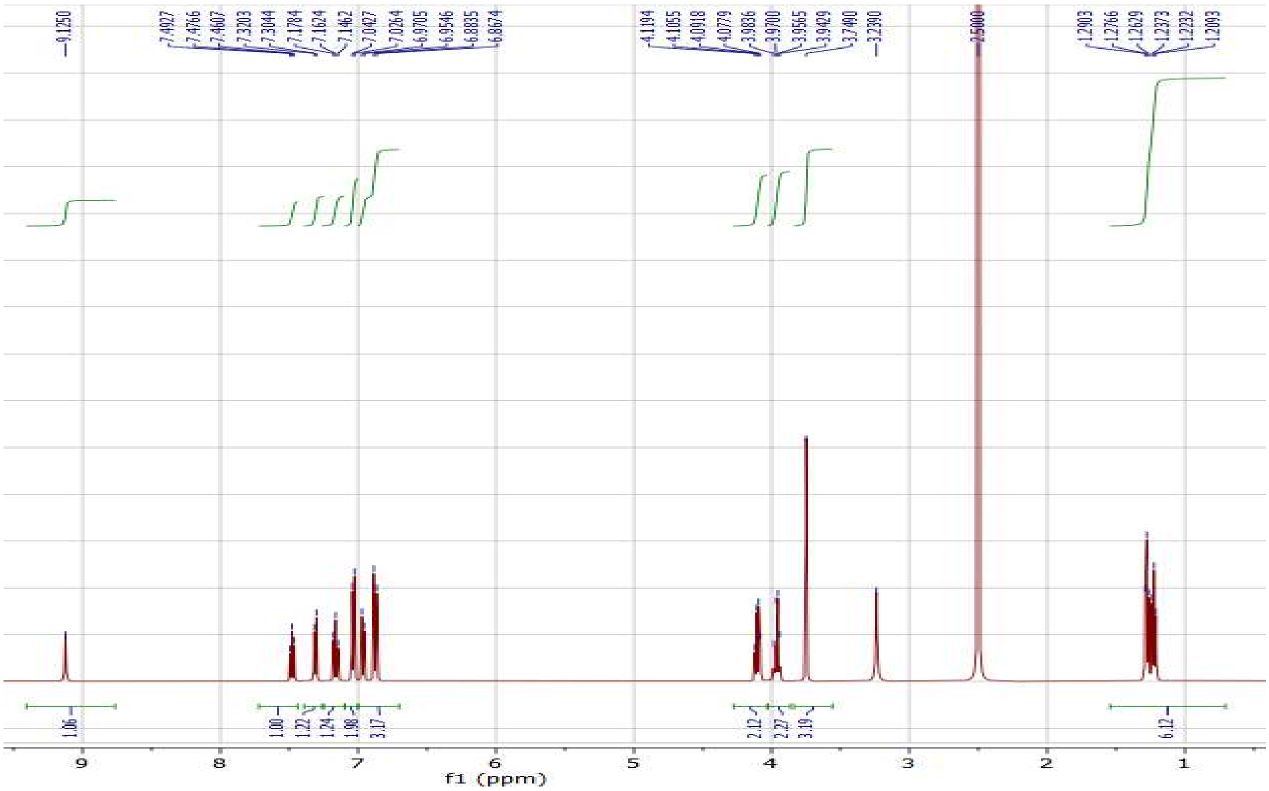

Figure S7. ^{1}H NMR spectrum of **4a** in DMSO-d$_6$ in DMSO-d$_6$

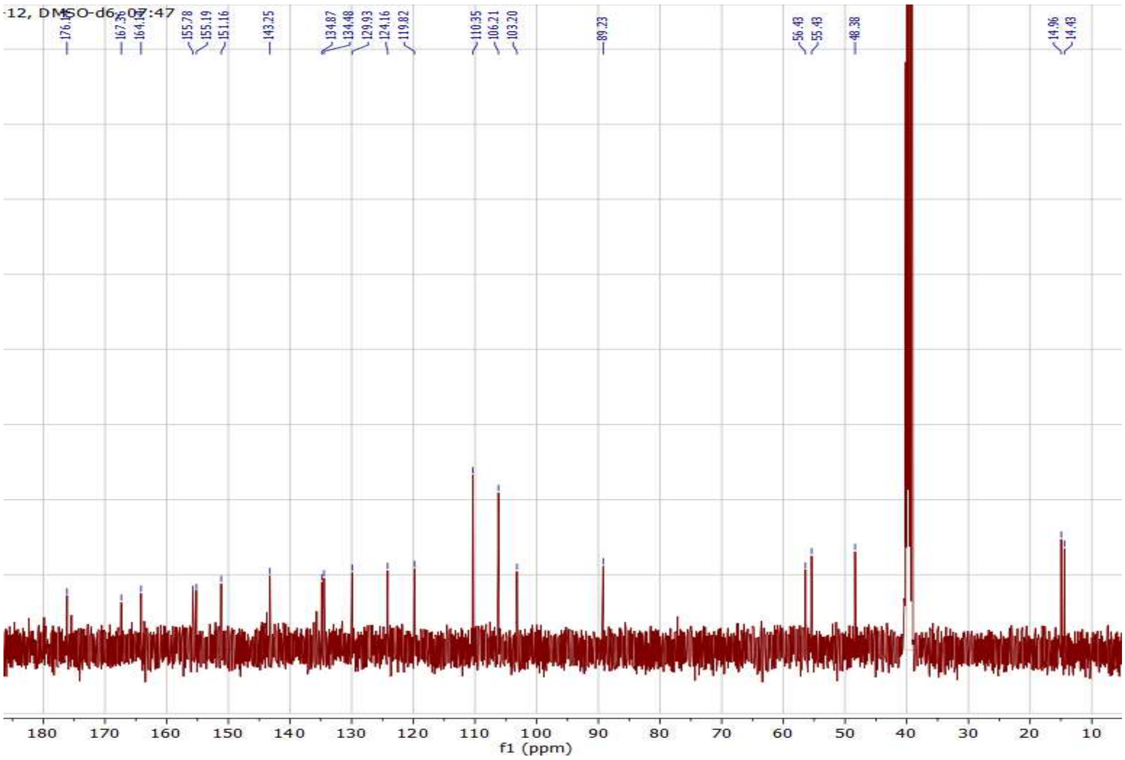

Figure S8. ^{13}C NMR spectrum of **4a** in DMSO-d$_6$

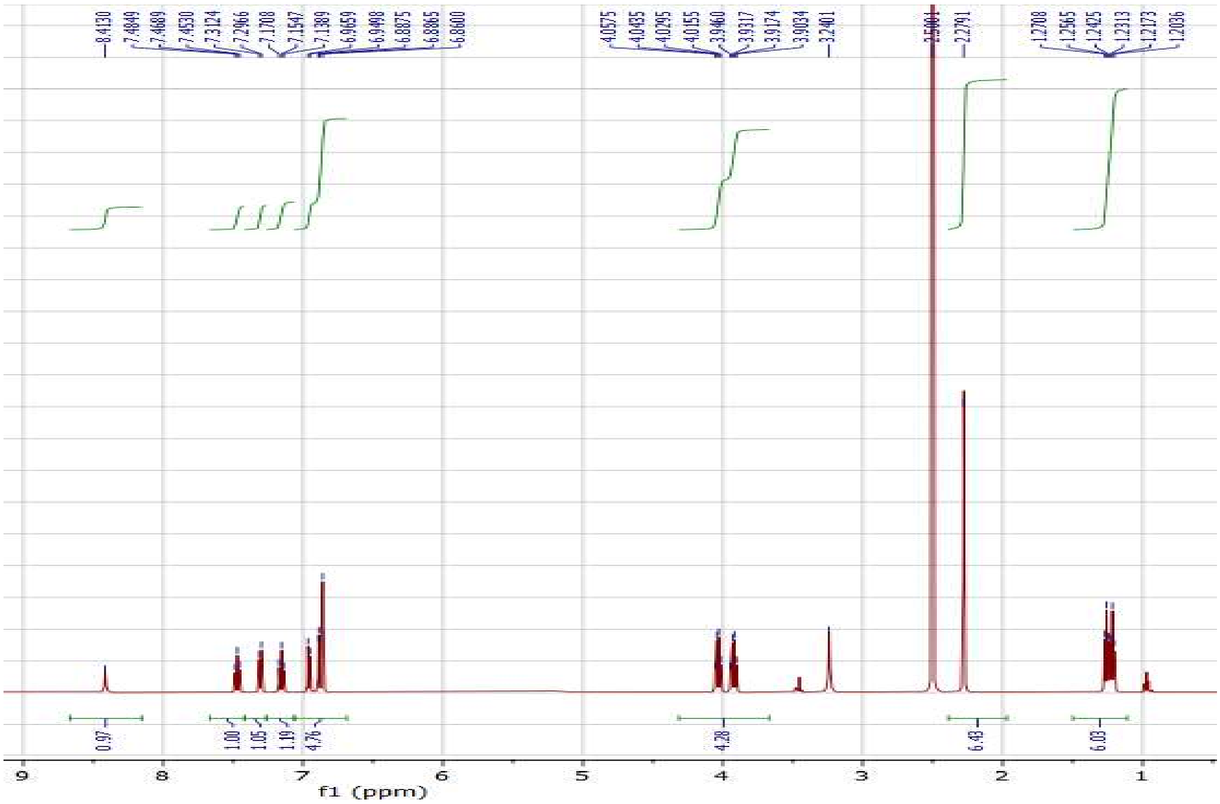

Figure S9. ^{1}H NMR spectrum of **5a** in DMSO-d_6

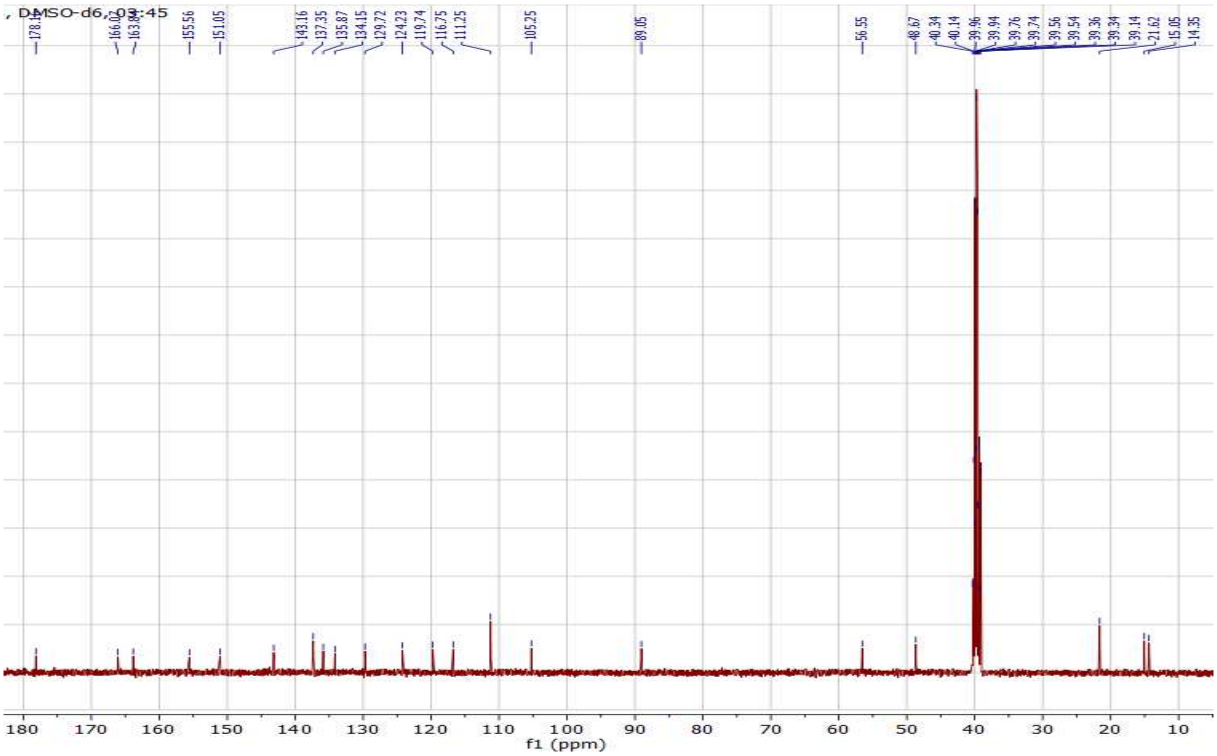

Figure S10. ^{13}C NMR spectrum of **5a** in DMSO-d_6

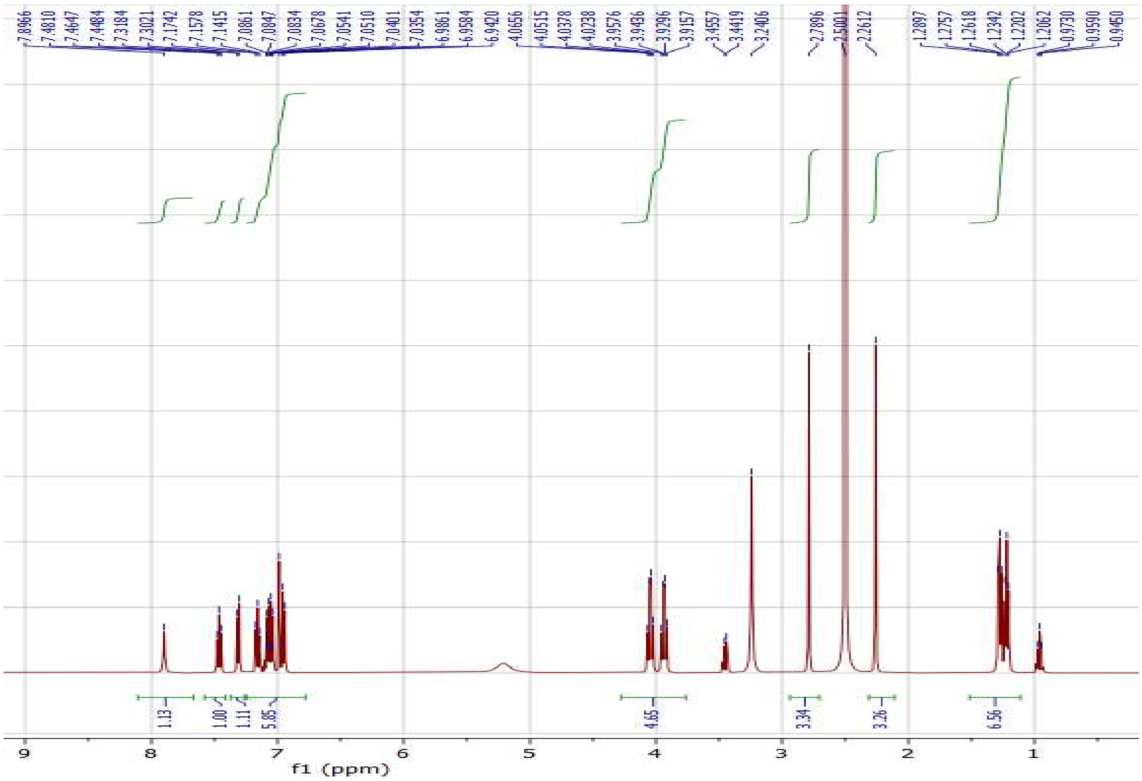

Figure S11. ^{1}H NMR spectrum of **6a** in DMSO-d_6

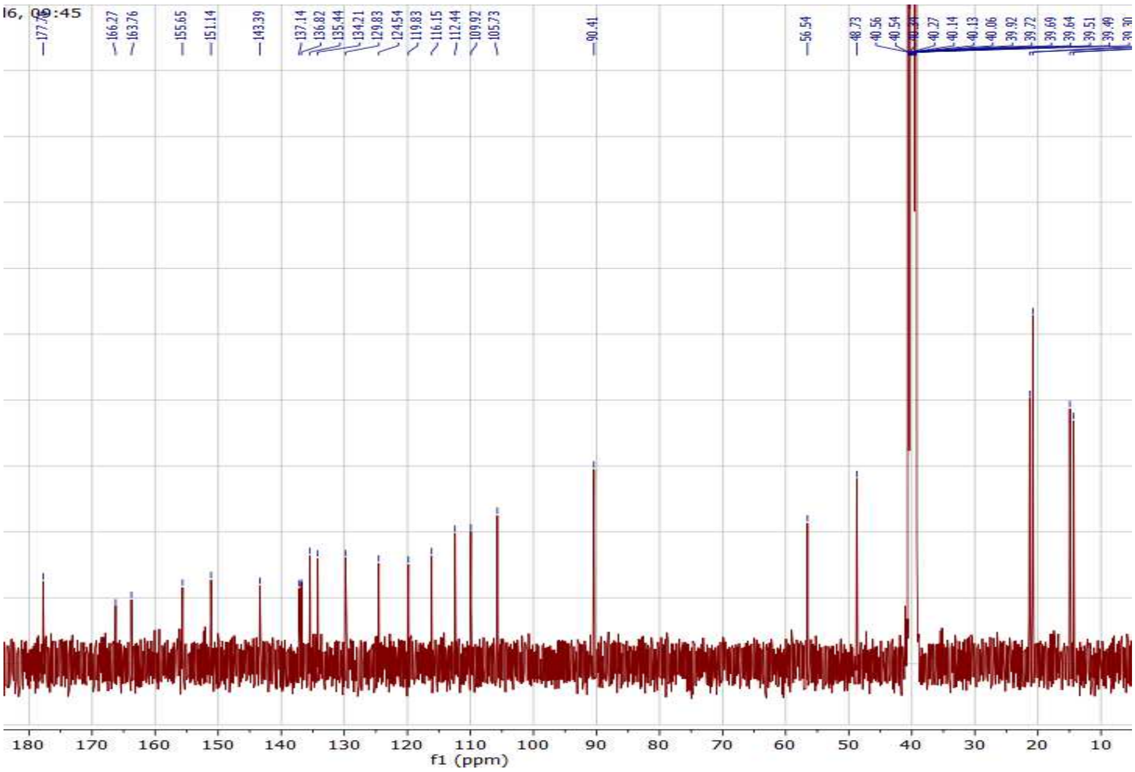

Figure S12. ^{13}C NMR spectrum of **6a** in DMSO-d_6

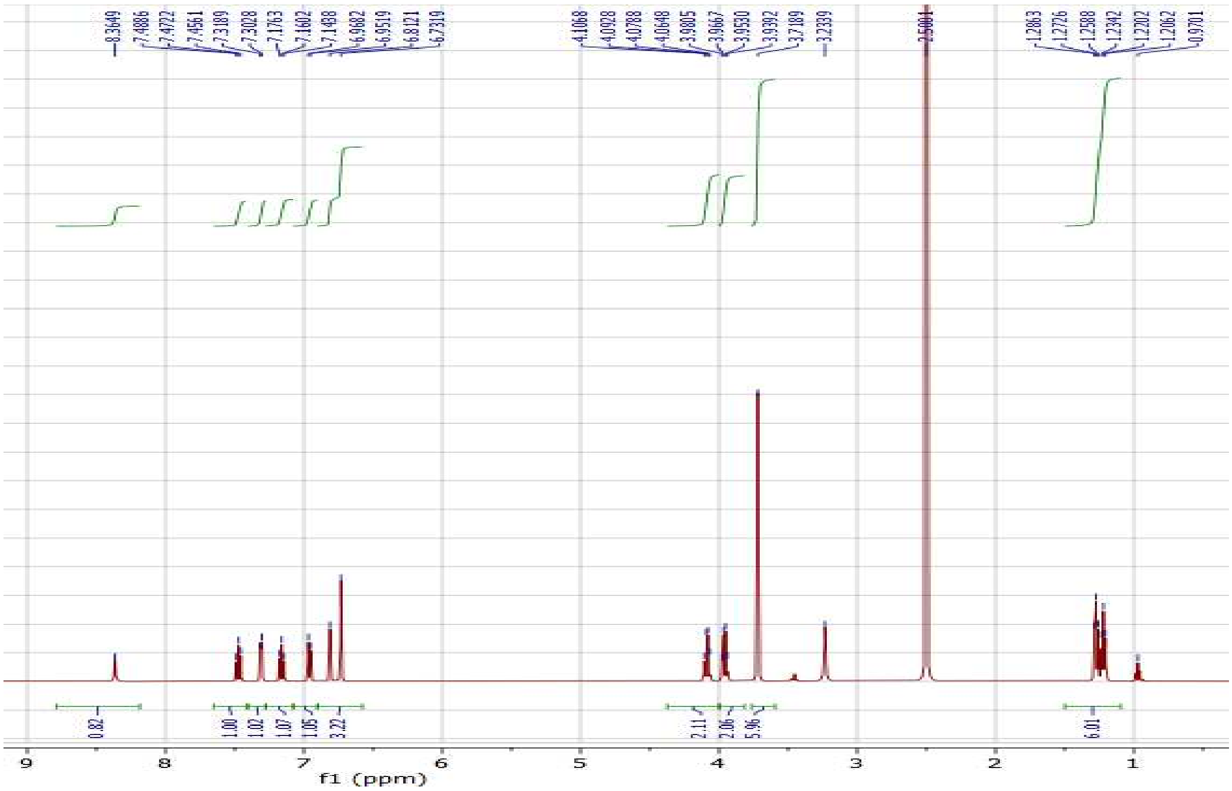

Figure S13. ^{1}H NMR spectrum of **7a** in DMSO-d_6

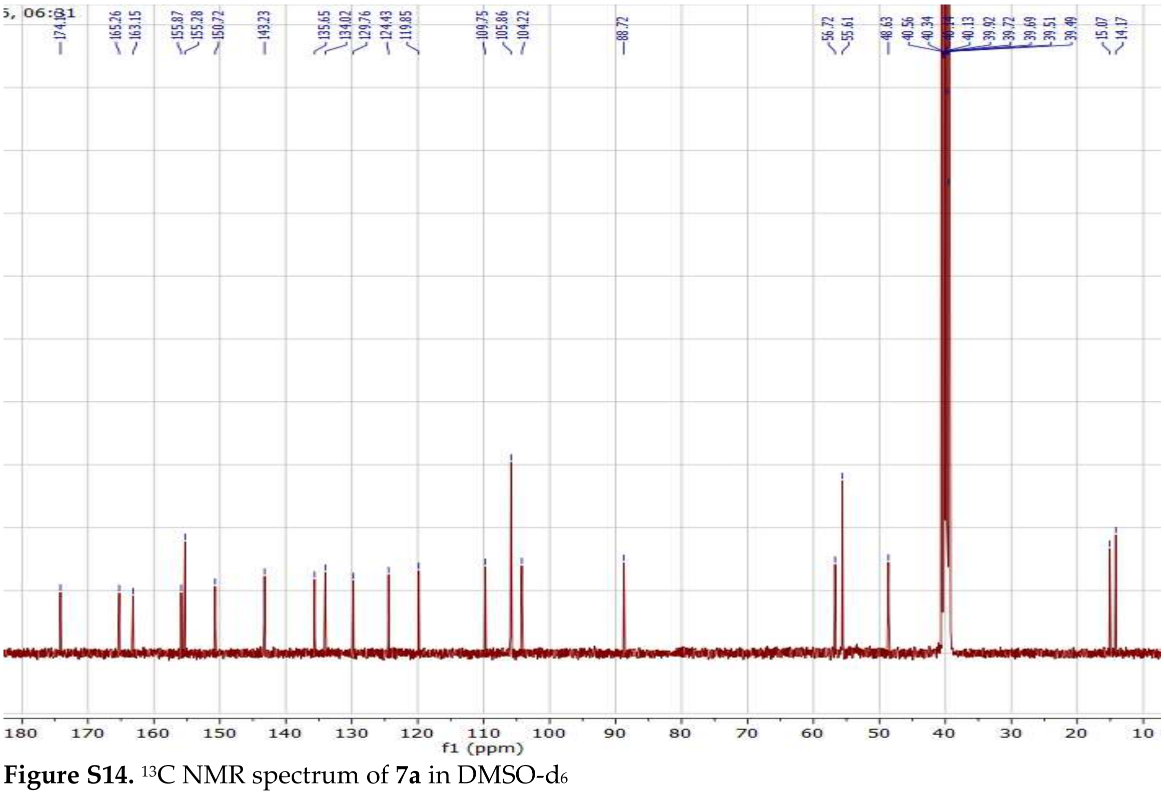

Figure S14. ^{13}C NMR spectrum of **7a** in DMSO-d_6

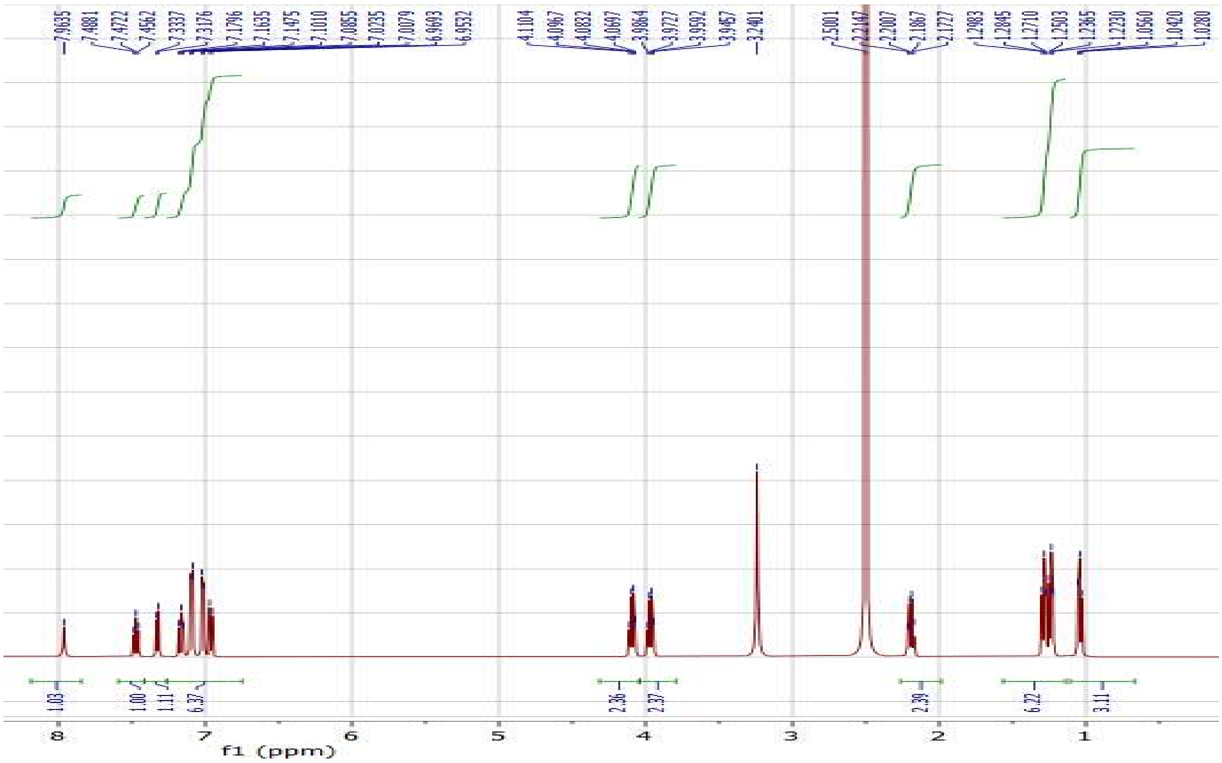

Figure S15. ^{1}H NMR spectrum of **8a** in DMSO-d_6

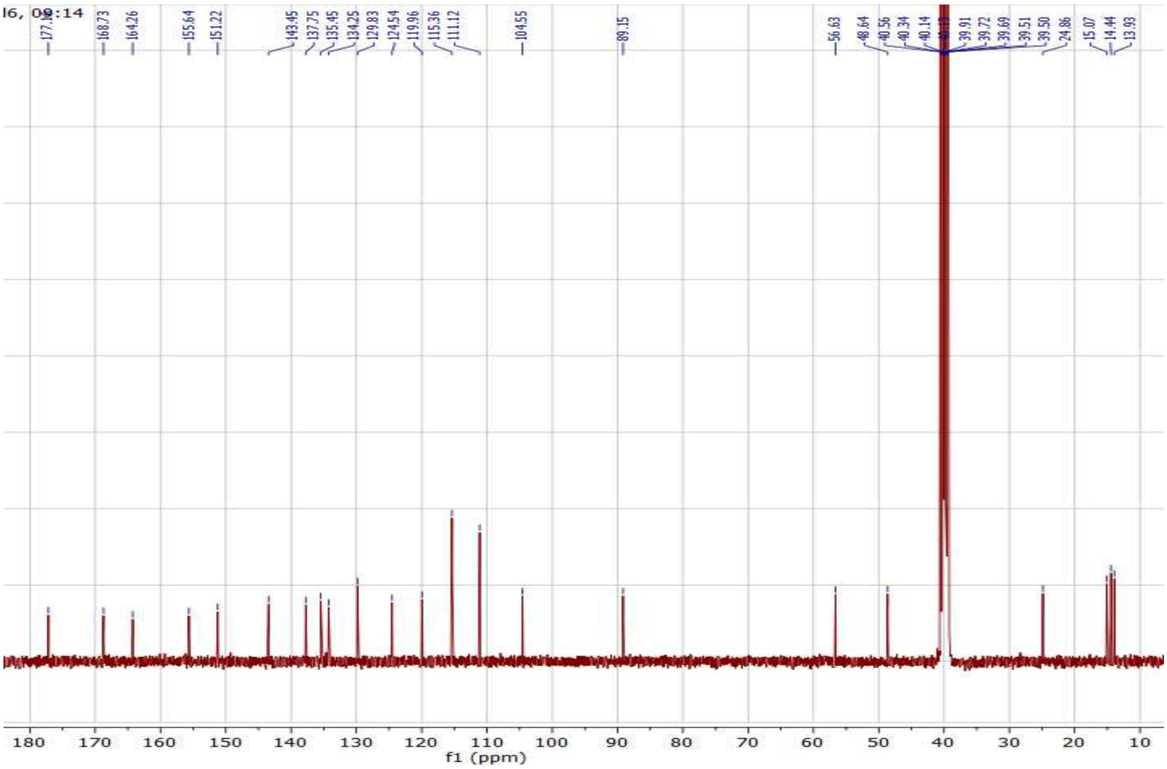

Figure S16. ^{13}C NMR spectrum of **8a** in DMSO-d_6

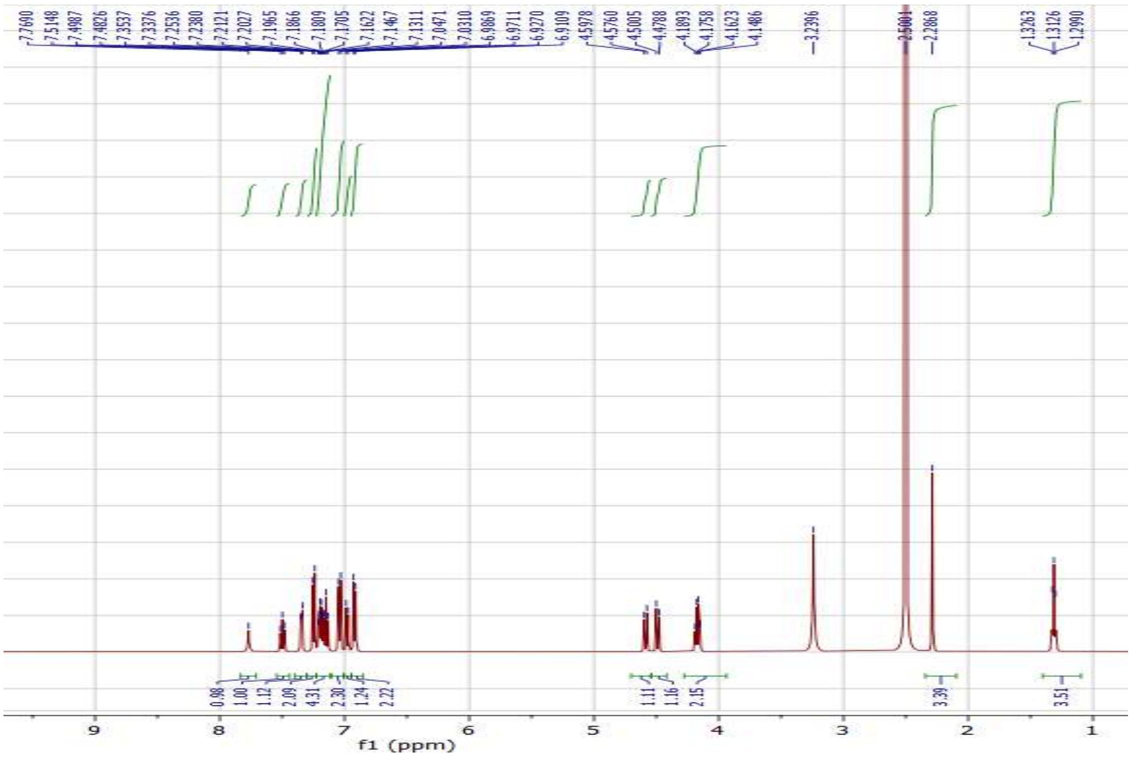

Figure S17. ^{1}H NMR spectrum of **9a** in DMSO-d_6

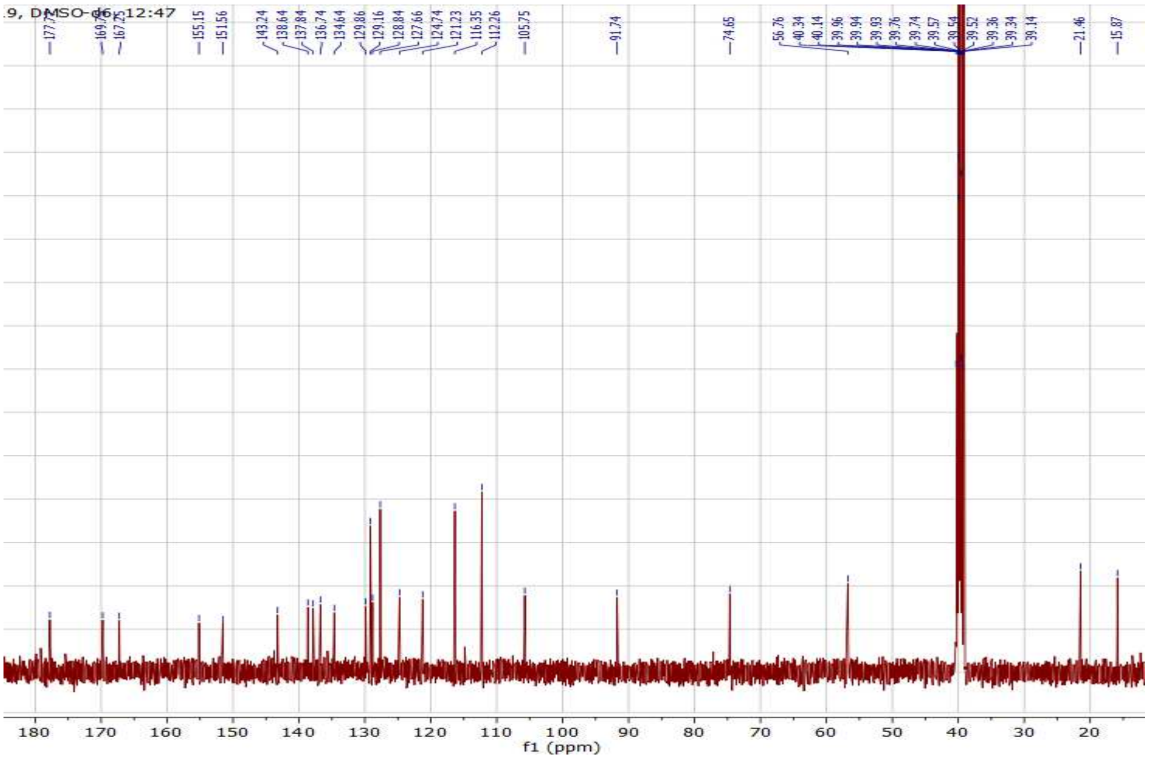

Figure S18. ^{13}C NMR spectrum of **9a** in DMSO-d_6

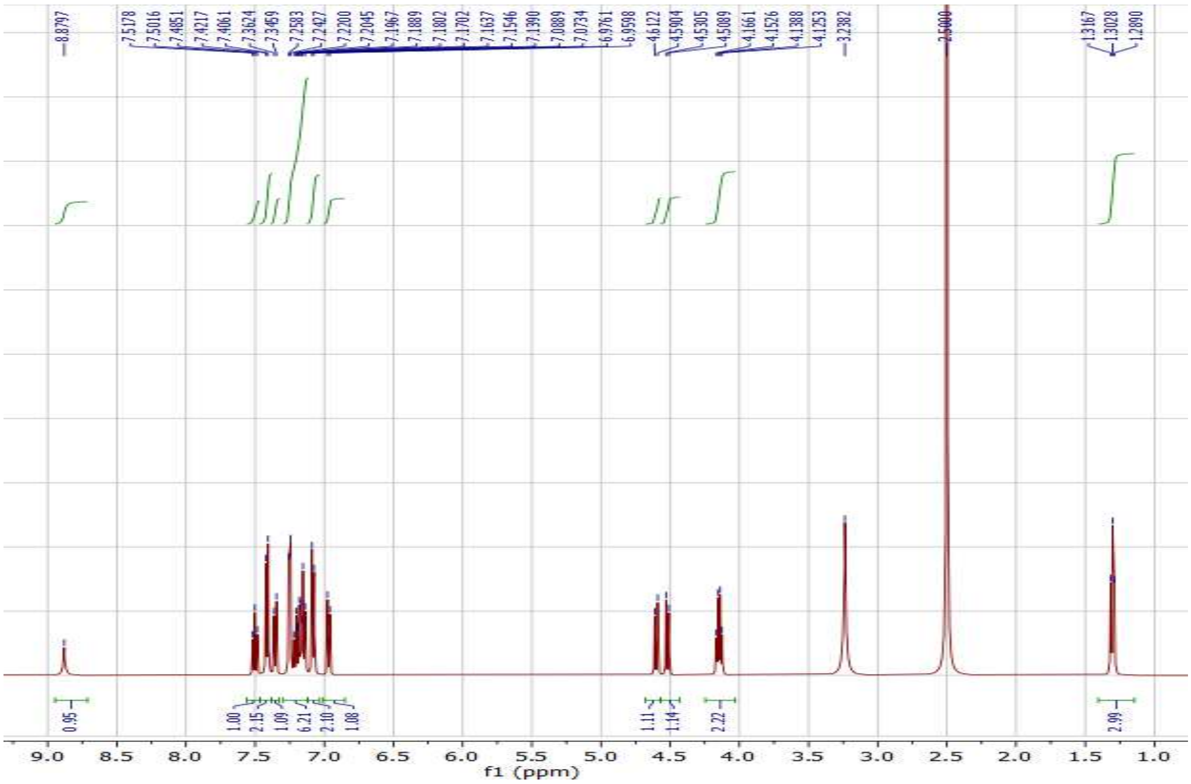

Figure S19. ^{1}H NMR spectrum of **10a** in DMSO-d_6

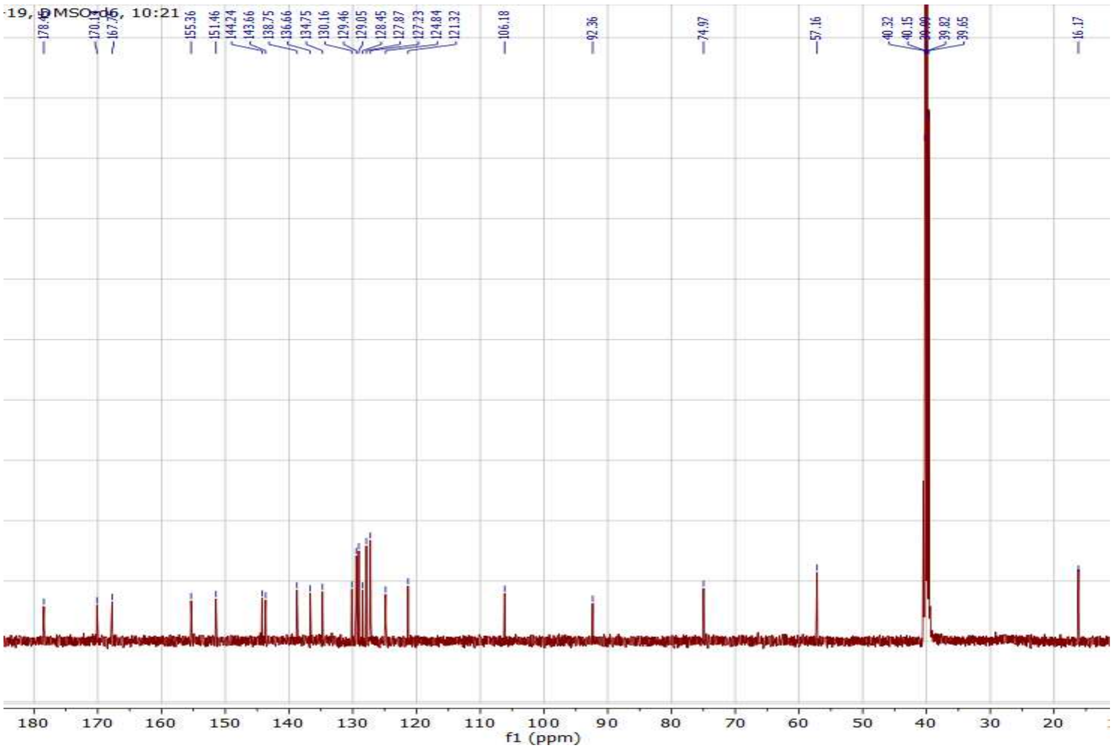

Figure S20. ^{13}C NMR spectrum of **10a** in DMSO-d_6

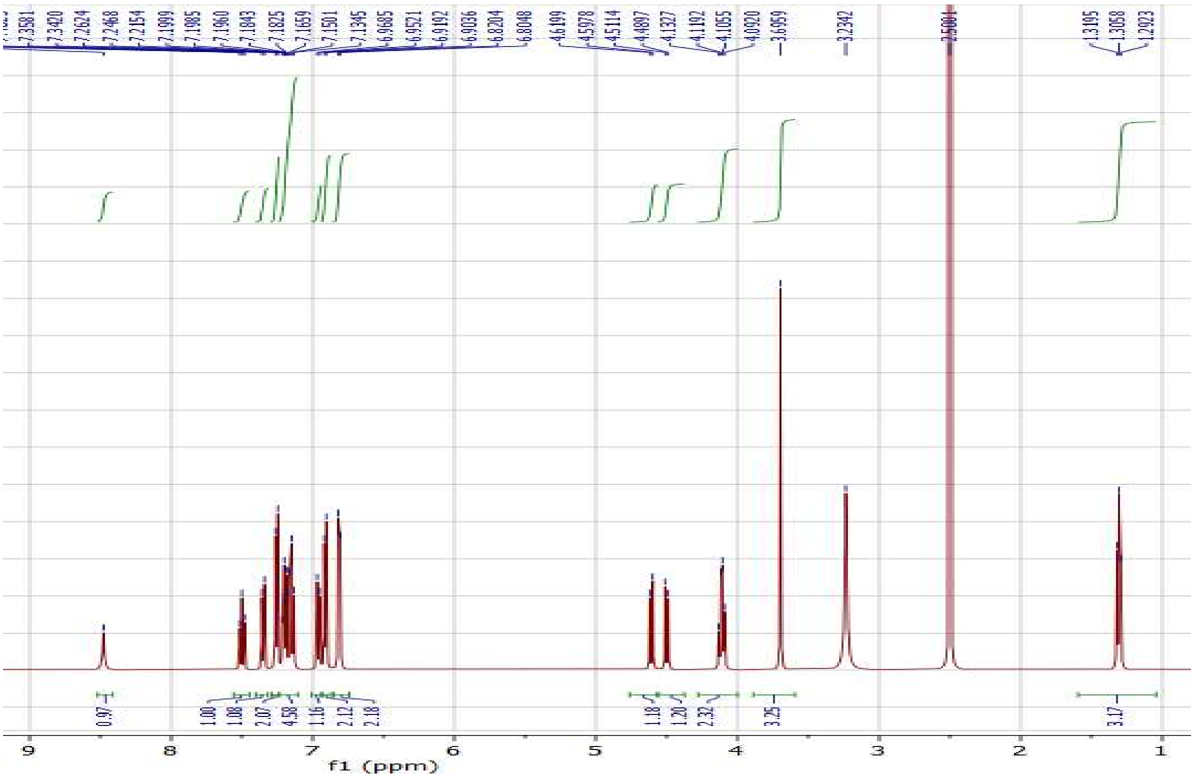

Figure S21. ^{1}H NMR spectrum of **11a** in DMSO-d_6

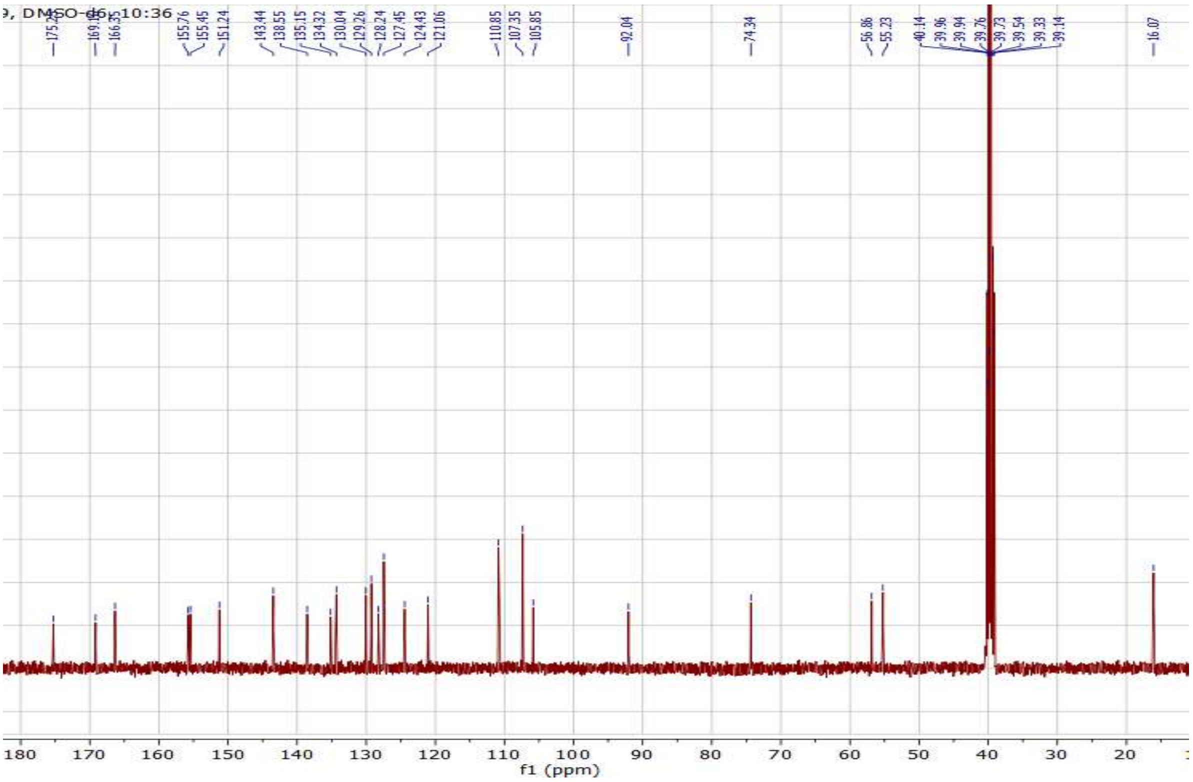

Figure S22. ^{13}C NMR spectrum of **11a** in DMSO-d_6

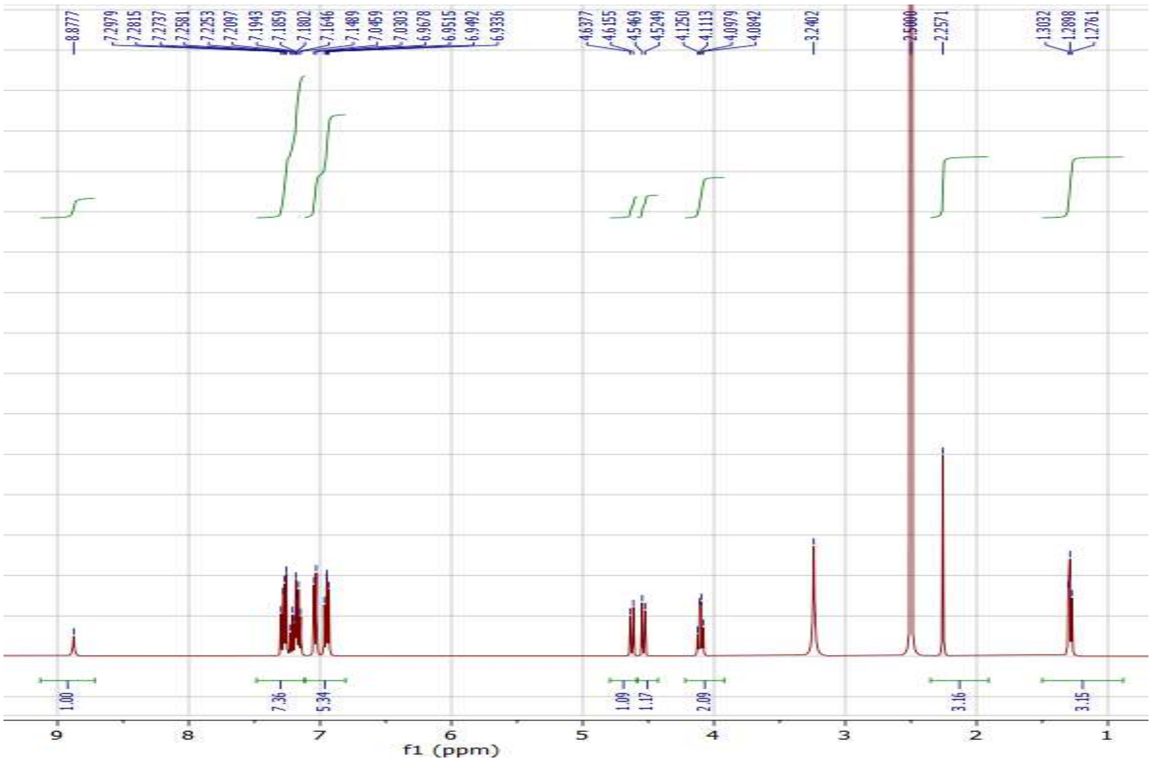

Figure S23. ^{1}H NMR spectrum of **12a** in DMSO-d$_6$

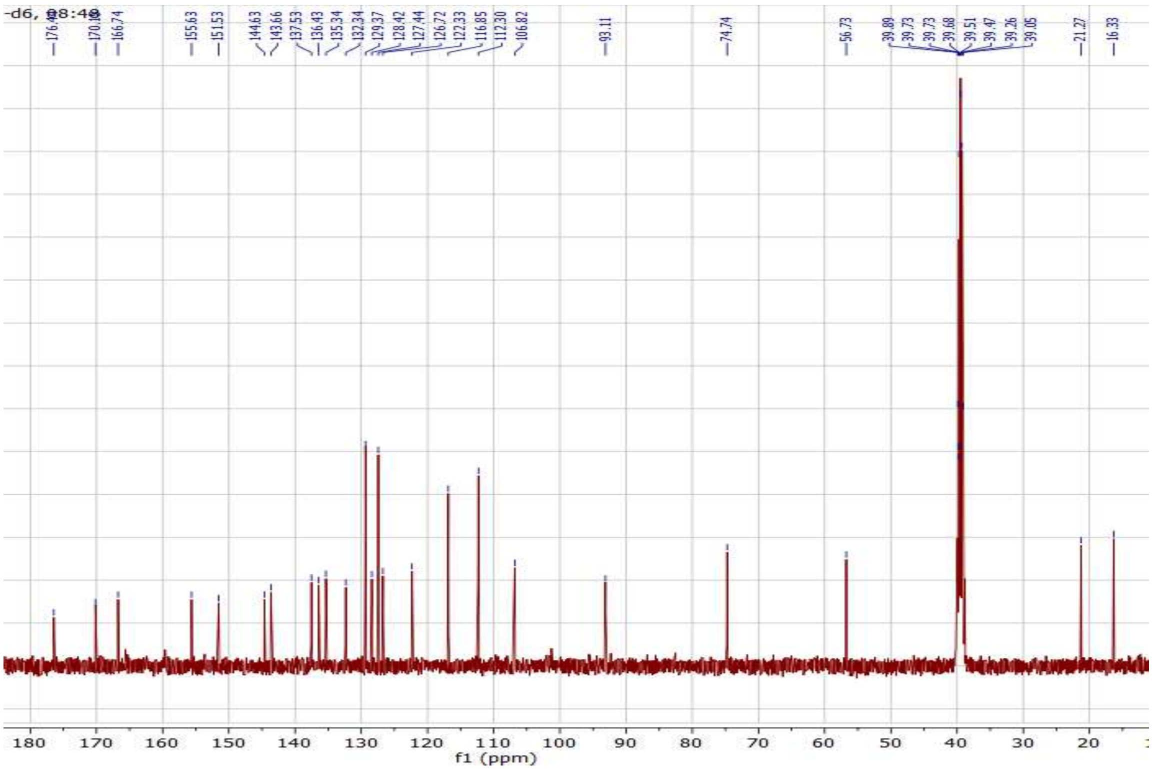

Figure S24. ^{13}C NMR spectrum of **12a** in DMSO-d$_6$

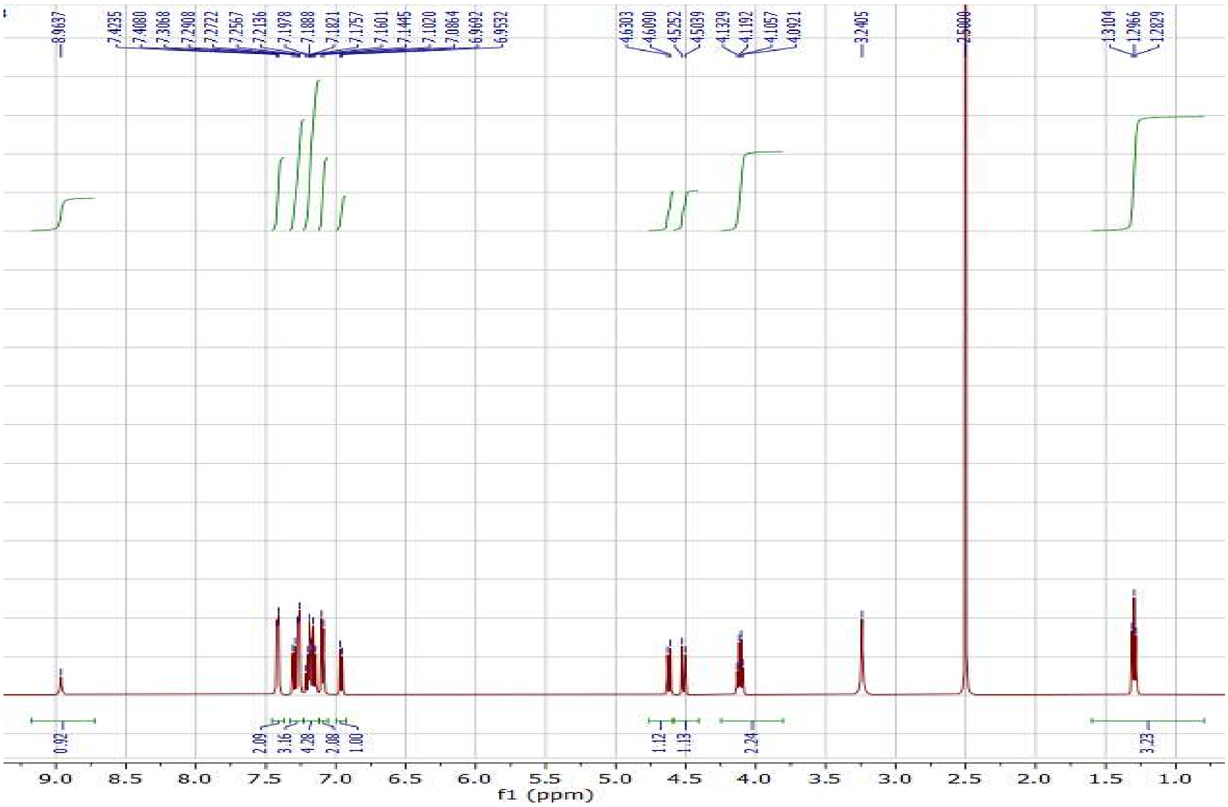

Figure S25. ^{1}H NMR spectrum of **13a** in DMSO-d$_6$

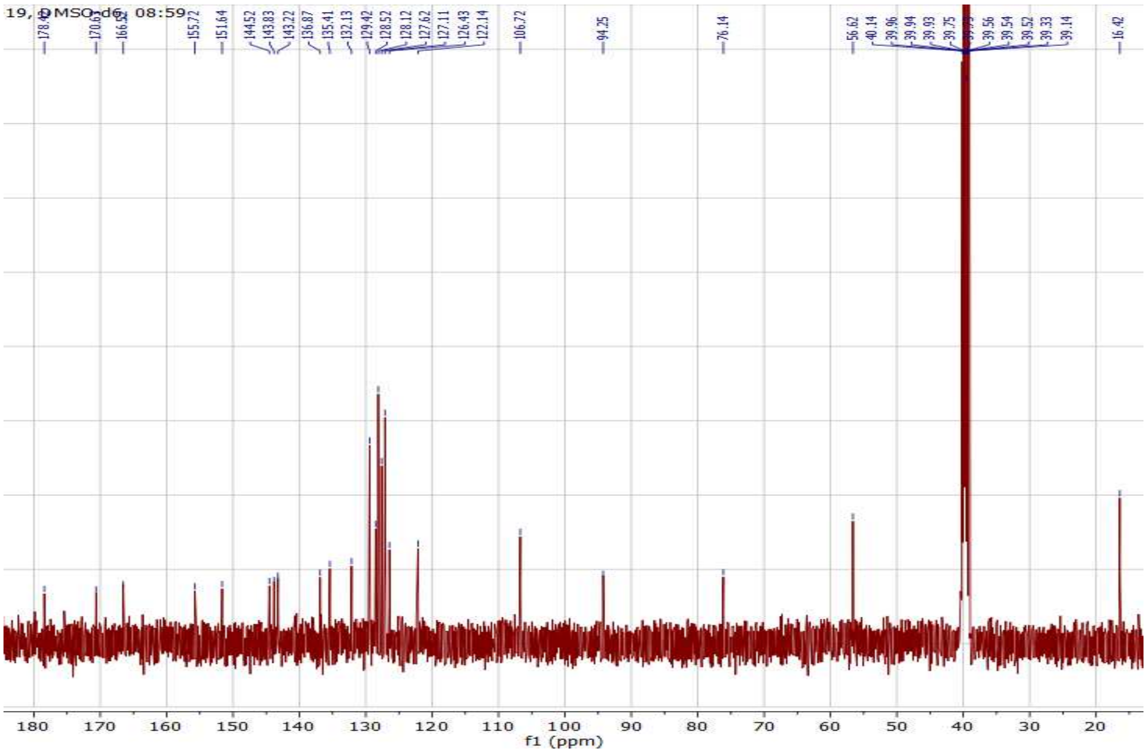

Figure S26. ^{13}C NMR spectrum of **13a** in DMSO-d$_6$

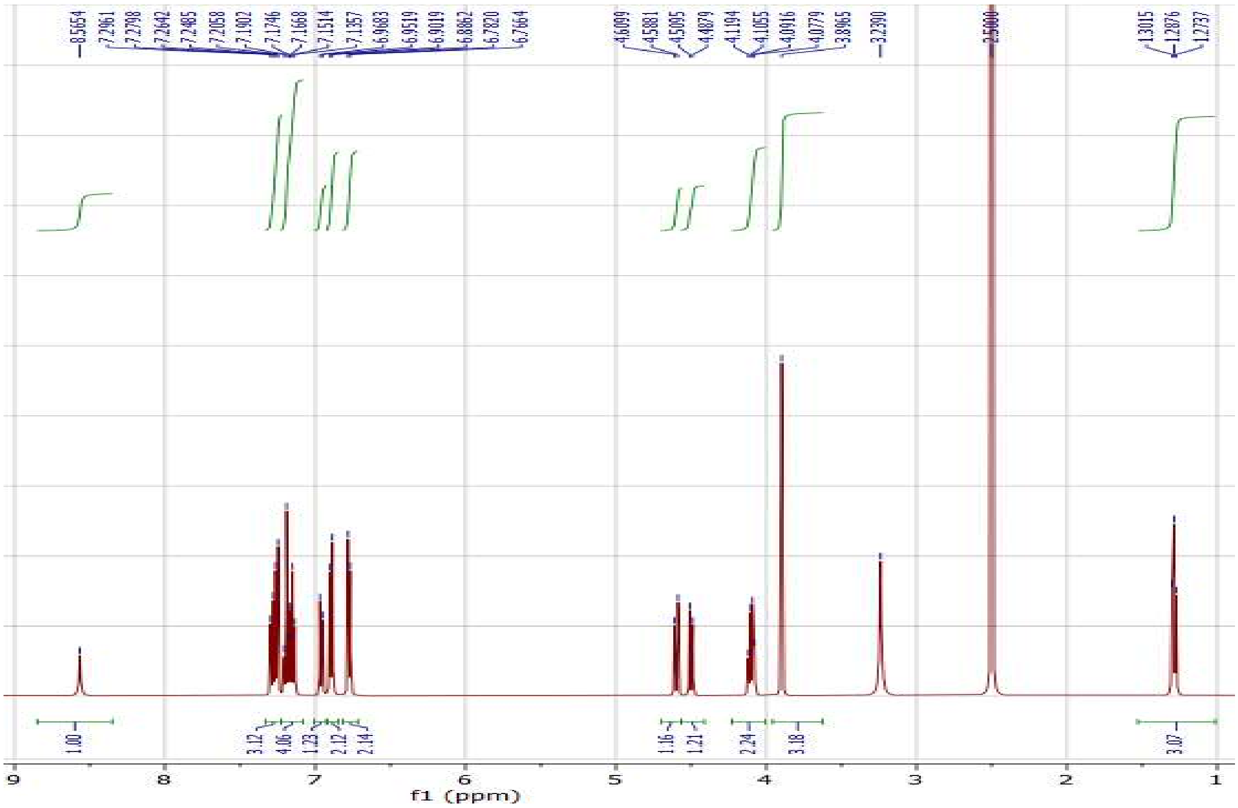

Figure S27. ^{1}H NMR spectrum of **14a** in DMSO-d_6

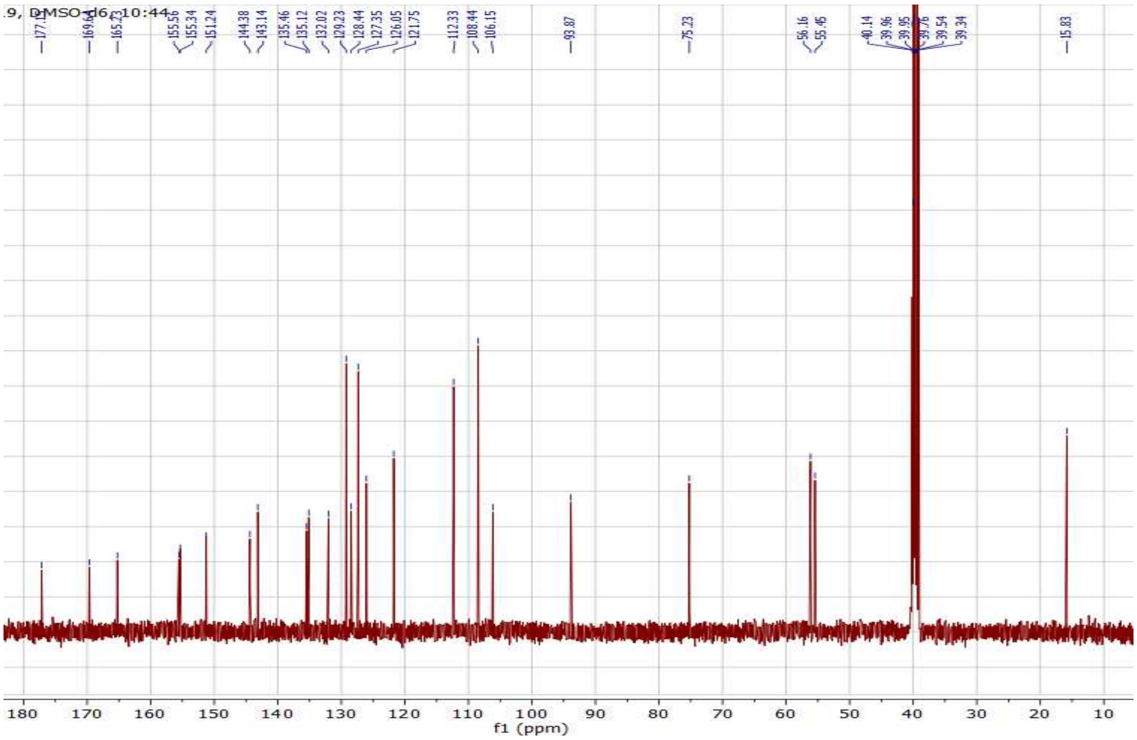

Figure S28. ^{13}C NMR spectrum of **14a** in DMSO-d_6

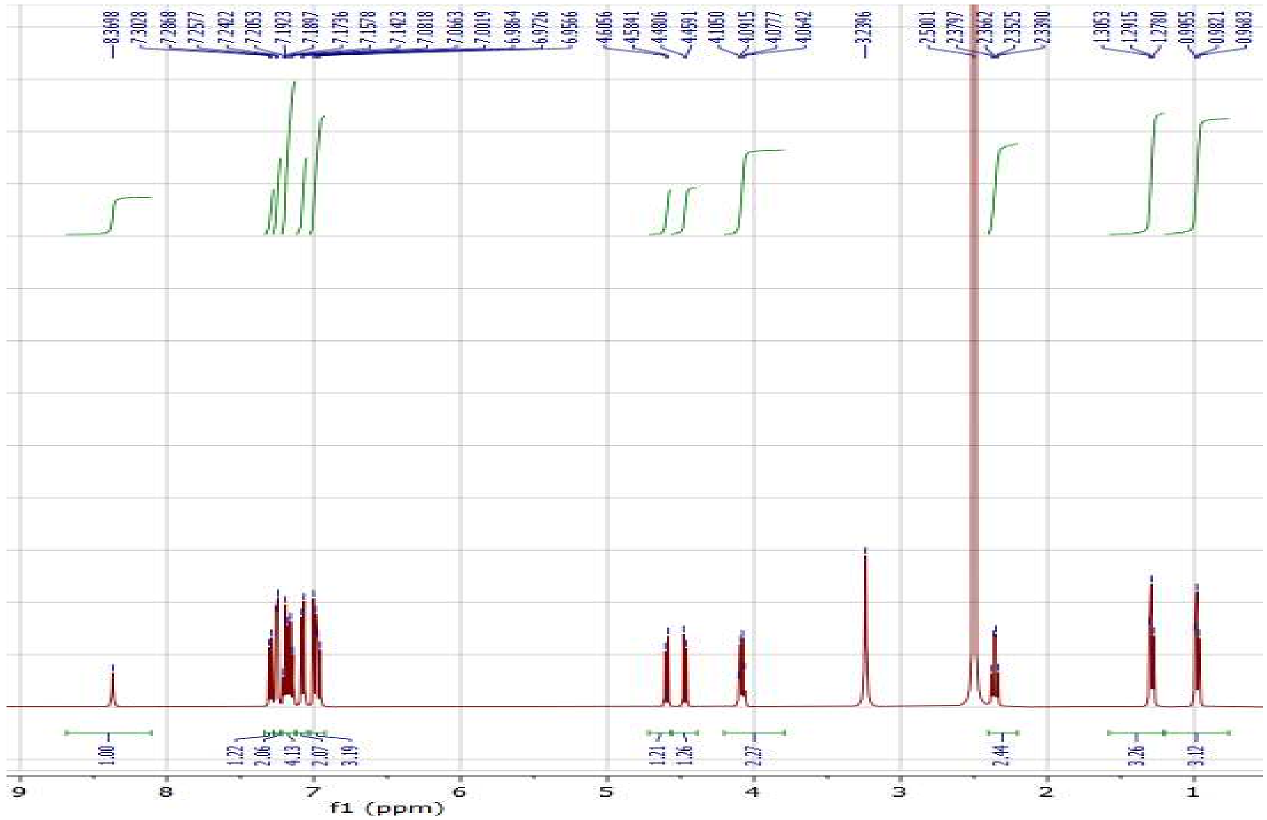

Figure S29. ^{1}H NMR spectrum of **15a** in DMSO-d$_6$

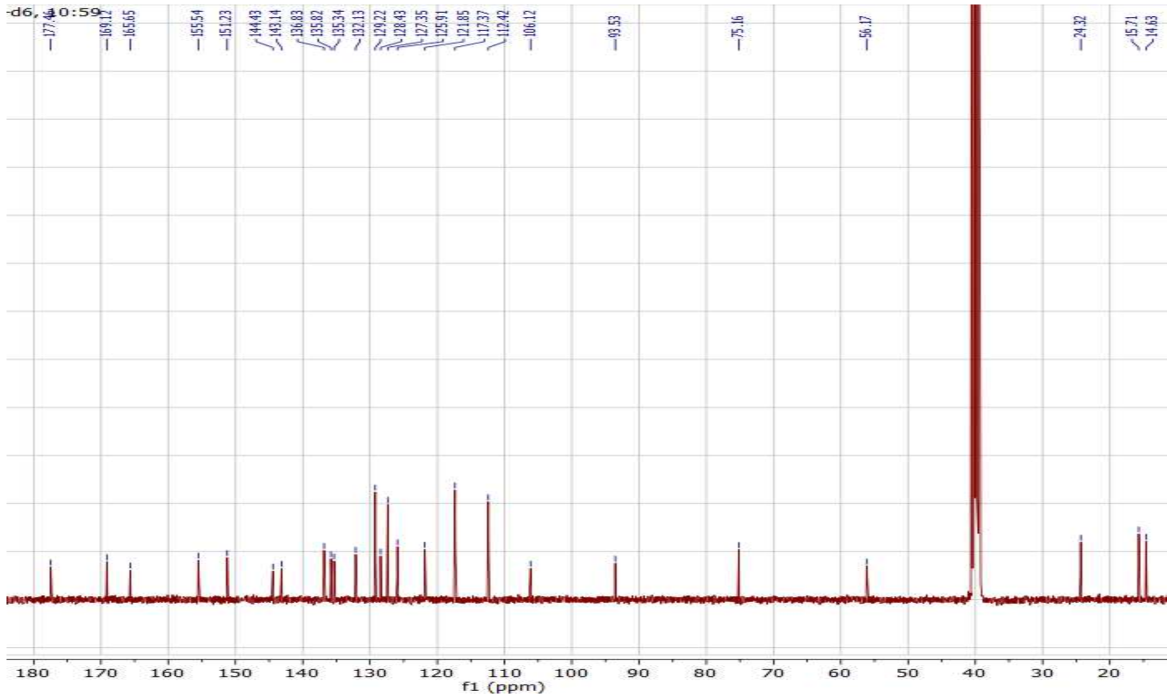

Figure S30. ^{13}C NMR spectrum of **15a** in DMSO-d$_6$

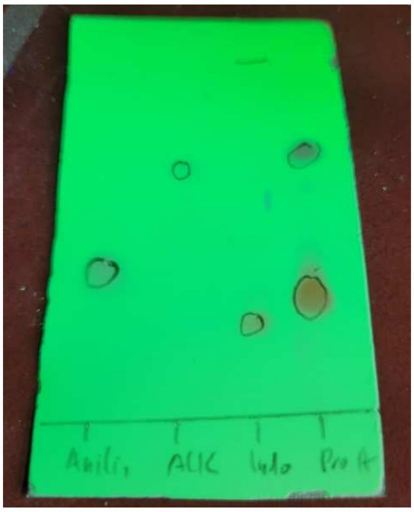

Figure S31. Photograph of the TLC control of the reaction of aniline (1. Trace from left to right), diethyl acetylenedicarboxylate (2. Trace), 1-ethylindoline-2-,3-dione (3. Trace), using HOAc as catalyst, with two spots of the product mixture (4. Trace). Conditions: 1:1:1 starting materials, 30 mol% HOAc, in 20 mL EtOH, 80°C, US, 3 h. TLC plates: silica, solvent: *c*-hexane/ethyl acetate (2/1).

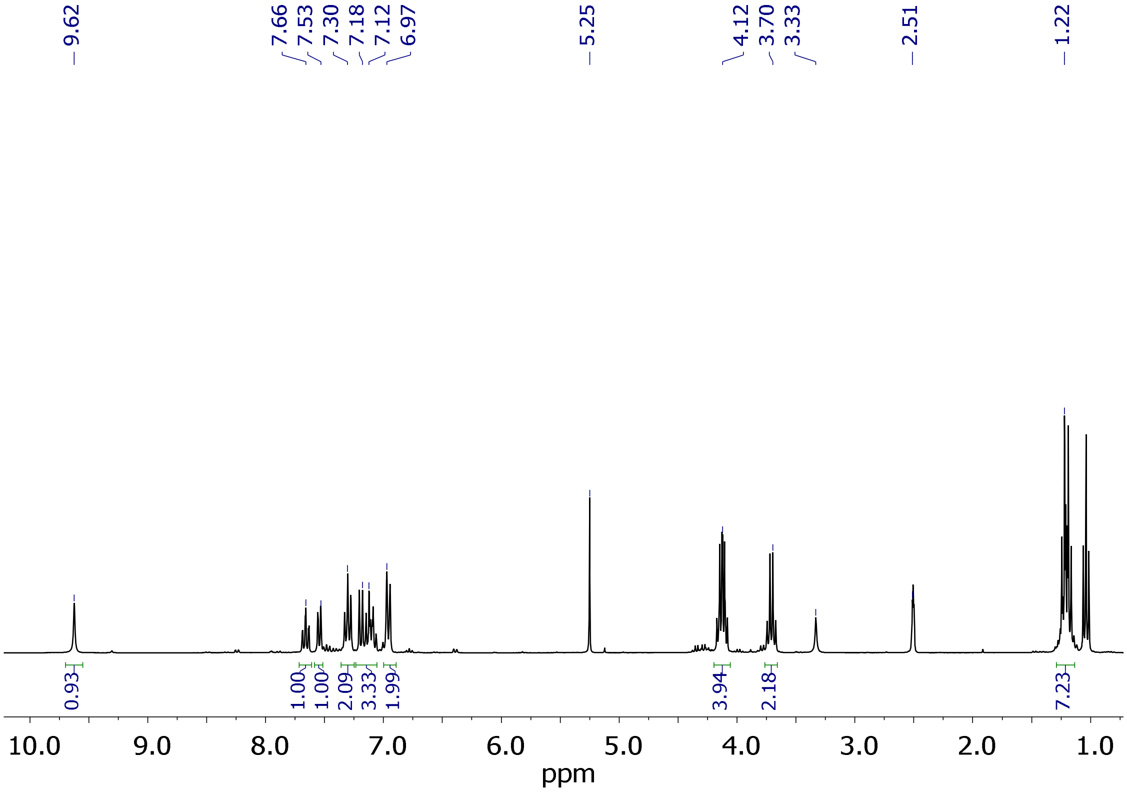

Figure S32. ^{1}H NMR spectrum of the product mixture from the reaction of aniline, diethyl acetylenedicarboxylate, 1-ethylindoline-2-,3-dione, using HOAc as catalyst, measured in DMSO-d_6. Conditions: 1:1:1 starting materials, 30 mol% HOAc, in 20 mL EtOH, 80°C, US, 3 h. The signals for the O-ethyl group of the target product **1a** are expected to be merged to a multiplet at 1.26 ppm, the NH proton is expected at 9.00 ppm (compare **Figure S1**).

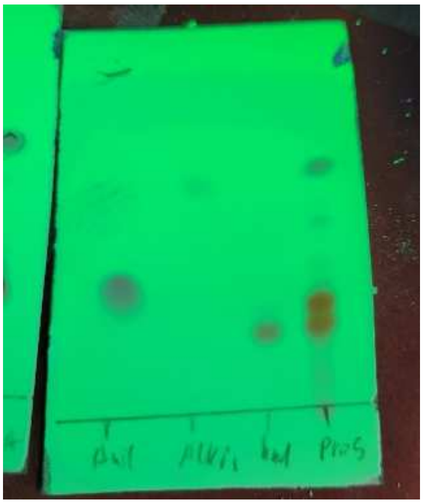

Figure S33. Photograph of the TLC control of the reaction of aniline (1. Trace from left to right), diethyl acetylenedicarboxylate (2. Trace), 1-ethylindoline-2-,3-dione (3. Trace), using H_2SO_4 as catalyst, with two spots of the product mixture (4. Trace). Conditions: 1:1:1 starting materials, 30 mol% H_2SO_4, in 20 mL EtOH, 80°C, US, 3 h. TLC plates: silica, solvent: *c*-hexane/ethyl acetate (2/1).

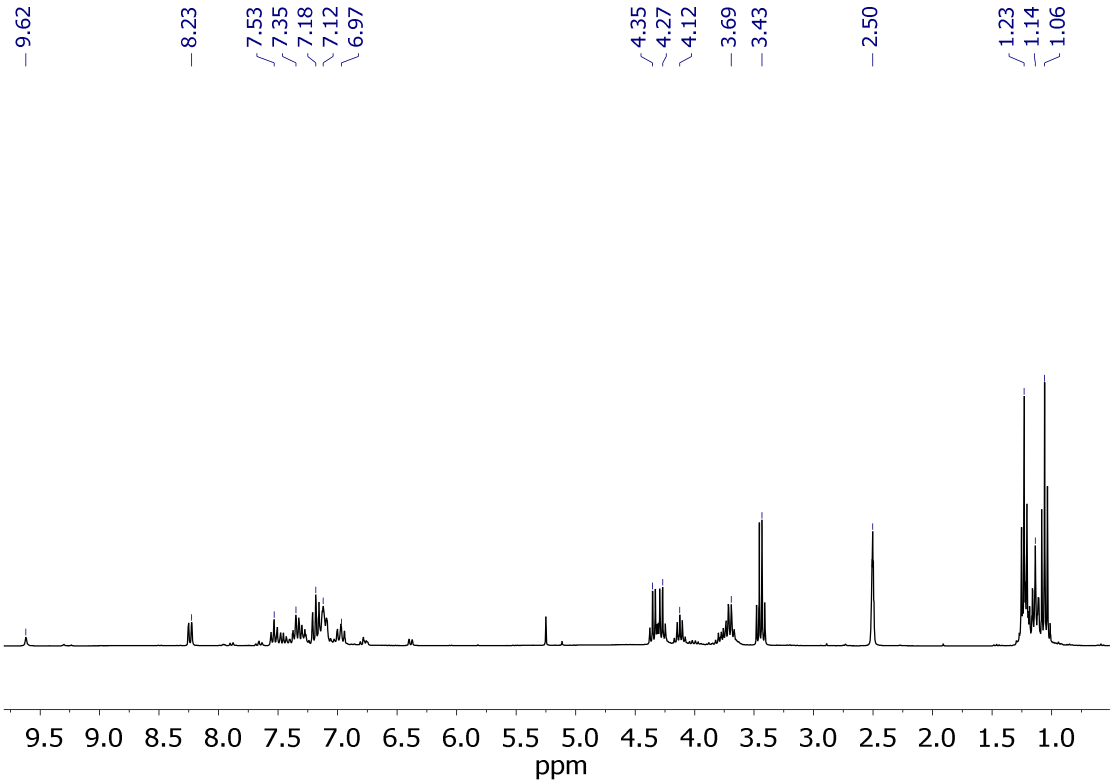

Figure S34. ^1H NMR spectrum of the product mixture from the reaction of aniline, diethyl acetylenedicarboxylate, 1-ethylindoline-2-,3-dione, using HOAc as catalyst, measured in DMSO-d_6. Conditions: 1:1:1 starting materials, 30 mol% HOAc, in 20 mL EtOH, 80°C, US, 3 h. The signals for the O-ethyl group of the target product **1a** are expected to be merged to a multiplet at 1.26 ppm, the NH proton is expected at 9.00 ppm (compare **Figure S1**).
